# Spinal cord elongation enables proportional regulation of the zebrafish posterior body

**DOI:** 10.1242/dev.204438

**Published:** 2025-01-09

**Authors:** Dillan Saunders, Carlos Camacho-Macorra, Benjamin Steventon

**Affiliations:** Department of Genetics, University of Cambridge, Cambridge, UK, CB2 3EH

**Keywords:** Zebrafish, Neuromesodermal progenitors, Mechanics, Morphogenesis, Robustness

## Abstract

Early embryos display a remarkable ability to regulate tissue patterning in response to changes in tissue size. However, it is not clear whether this ability continues into post-gastrulation stages. Here, we performed targeted removal of dorsal progenitors in the zebrafish tailbud using multiphoton ablation. This led to a proportional reduction in the length of the spinal cord and paraxial mesoderm in the tail, revealing a capacity for the regulation of tissue morphogenesis during tail formation. Following analysis of cell proliferation, gene expression, signalling and cell movements, we found no evidence of cell fate switching from mesoderm to neural fate to compensate for neural progenitor loss. Furthermore, tail paraxial mesoderm length is not reduced upon direct removal of an equivalent number of mesoderm progenitors, ruling out the hypothesis that neuromesodermal competent cells enable proportional regulation. Instead, reduction in cell number across the spinal cord reduces both spinal cord and paraxial mesoderm length. We conclude that spinal cord elongation is a driver of paraxial mesoderm elongation in the zebrafish tail and that this can explain proportional regulation upon neural progenitor reduction.

## Teaser

**Highlighted Article:** Targeted ablation of dorsal progenitors in the zebrafish tailbud shows how the rate of spinal cord elongation governs the corresponding elongation of the neighbouring paraxial mesoderm.

## INTRODUCTION

Cells must coordinate their morphogenesis, differentiation, and growth to form embryonic tissues. In turn, tissue expansion must be coordinated such that each tissue forms with the correct proportions for the embryo. Investigations into the control of tissue proportions have predominantly focused on the specification of their primordia from a field of competent cells. Many of these patterning signals show a remarkable ability to scale with changes to the size of the field and have been thoroughly reviewed elsewhere (Čapek and Müller, 2019; Thompson et al., 2018). Tissue proportions can also be coordinated after their specification through the coupling of the morphogenesis of a tissue with that of its neighbours. Although less studied, this type of proportional regulation has been recently described in the elongation of the body axis in zebrafish (McLaren and Steventon, 2021; Tlili et al., 2019) and avian (Romanos et al., 2024; Xiong et al., 2020) embryos. This phenomenon is an example of multi-tissue tectonics, in which the deformation of a tissue at the mesoscopic scale, known as tissue tectonics (Blanchard et al., 2009), can impact the dynamics of morphogenesis in neighbouring tissues (Busby and Steventon, 2021).

The ability of an embryo to regulate the proportions of its tissues when reduced in size is most evident prior to, and during gastrulation, and has been demonstrated in zebrafish (Almuedo-Castillo et al., 2018; Ishimatsu et al., 2018; Huang and Umulis, 2019), *Xenopus* (De Robertis, 2009), chick (Spratt and Haas, 1960) and mouse (Nichols et al., 2022). In the case of fish and frog embryos, the resulting correctly patterned body plan does not recover its wild-type size, whereas some species such as mouse display both proportional and absolute size regulation (Nichols et al., 2022).

The formation and morphogenesis of the primary embryonic body axes occurs during gastrulation and then continues through a process known as posterior body elongation, which generates the embryo's tail and a species-specific amount of its trunk (Steventon and Martinez Arias, 2017). The elongation of the tail tissues, such as spinal cord and paraxial mesoderm [somites and pre-somitic mesoderm (PSM)], occurs through a combination of progenitor addition and subsequent morphogenesis of the tail tissues (Bénazéraf, 2019; Steventon et al., 2016). The raw materials for tissue extension are the progenitor cells located most caudally in the embryo in a structure known as the tailbud. There is now considerable evidence that some of these progenitor cells, often known as neuromesodermal progenitors (NMPs), are not restricted to either neural or mesodermal identity and can give rise to descendants that end up in either tissue type. The details of this have been reviewed extensively elsewhere (Wymeersch et al., 2021).

In zebrafish, the manipulation of Wnt signalling transduction in single cells determines whether they become neural or mesodermal (Martin and Kimelman, 2012) and from this we can infer that neuro-mesodermal competent cells are present in the tailbud. However, cell tracking at various developmental stages has found few or no progenitors producing both neural and mesodermal descendants (Attardi et al., 2018; Kanki and Ho, 1997; Lange et al., 2024). This is because NMP behaviour is dependent on the environment of the cells. For example, the low levels of cell division in the zebrafish tailbud (Steventon et al., 2016; Attardi et al., 2018; Kanki and Ho, 1997; Bouldin et al., 2014) mean that cells have few progeny to contribute to either tissue type. This has been proposed to account for differences in NMP behaviour between mouse and zebrafish (Wymeersch et al., 2021; Sambasivan and Steventon, 2021). Importantly, another factor that could affect NMP behaviour is the morphogenetic flow of the progenitor cells, which was first proposed to affect the prevalence of NMP cells in the early chick embryo as these cells are located at the divergence of two flows (Wood et al., 2019 preprint). Notably, a similar difference in morphogenetic behaviour appears to separate dorsal and ventral located progenitors in the zebrafish tailbud (Lange et al., 2024; Lawton et al., 2013). This has led to the proposal to use the broader term neuromesodermal competent cells (NMC cells) when referring to these progenitors (Binagui-Casas et al., 2021).

Given that this competent cell population is retained in the zebrafish tailbud without it being utilised as a stem cell pool, it has been suggested that this population may enable zebrafish embryos to be robust to imbalance of neural and mesoderm progenitor allocation during gastrulation (Sambasivan and Steventon, 2021). In line with this hypothesis, inhibition of Wnt signalling throughout the zebrafish embryo during this period leads to the loss of paraxial mesoderm but not the loss of neural tissue (Martin and Kimelman, 2012). However, there is a growing body of evidence that zebrafish posterior body elongation is driven primarily by the morphogenesis and growth of the tail tissues, anterior to the tailbud (McLaren and Steventon, 2021; Tlili et al., 2019; Steventon et al., 2016; Özelçi et al., 2022). Furthermore, studies have shown that these tissues are coupled together through the extracellular matrix (Tlili et al., 2019; Guillon et al., 2020). This raises the alternative hypothesis that multi-tissue tectonics, either through mechanical action or inter-tissue signalling, could ensure the proportional elongation of neural and paraxial mesodermal tissue.

Consequently, we set out to investigate whether there is any capacity for the proportional regulation of neural and mesodermal tissue elongation in the zebrafish tail, and, if so, to determine whether this regulation is coordinated by changes in NMC cell behaviour or multi-tissue tectonics.

## RESULTS

### Loss of dorsal progenitors results in a proportional reduction in tail elongation

To investigate whether there is a capacity for regulation of the proportion of neural and mesodermal tissue in the tail, we needed a method to reduce the number of cells in the system. We chose to use two-photon ablation as it allows the targeting of a subpopulation of cells in three dimensions and acts rapidly to cause cell death (Lanvin et al., 2015; Vogel et al., 2005). Additionally, two-photon ablation has recently been utilised to investigate the role of notochord growth in zebrafish tail development (McLaren and Steventon, 2021).

**Fig. 1.**
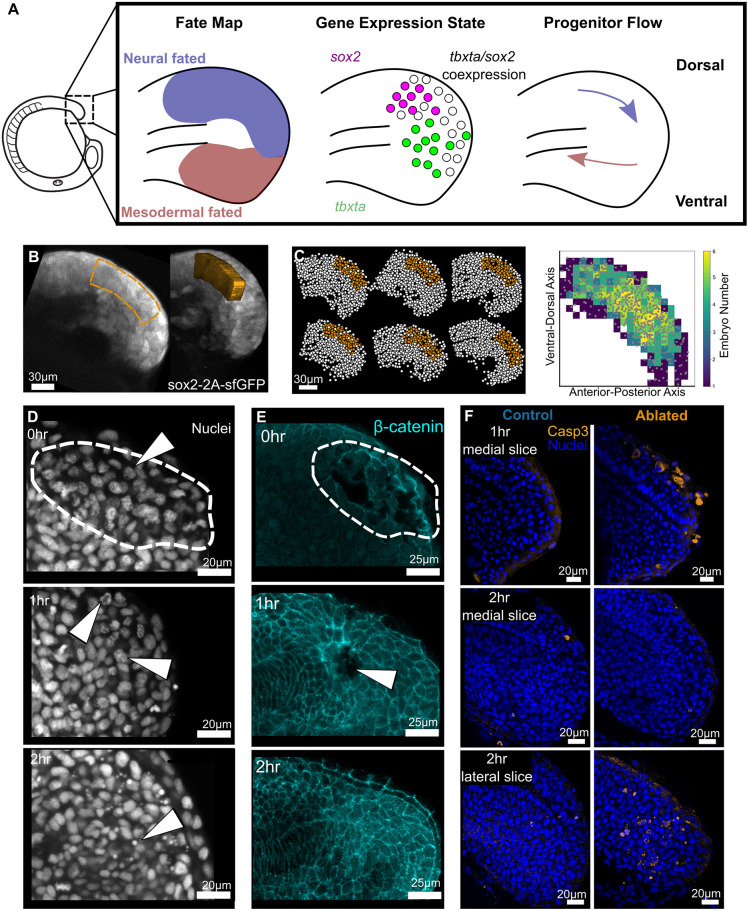
**Two-photon ablation causes localised cell death in the dorsal progenitor region.** (A) Diagram showing the zebrafish embryo at the 14-somite stage with an inset panel showing the general fate map, heterogeneous gene expression states, and predominant movement of progenitors in the dorsal and ventral regions of the tailbud. (B) Lateral and oblique views of a typical 3D ablation region of interest located in the dorsal progenitor region as indicated by Sox2:GFP expression. (C) Result of segmentation and registration of tailbud nuclei, with ablated region in orange. The precision of the ablation location between embryos is displayed in the heatmap (*n*=6) and shows a high level of precision for user-selected ROIs. (D) Representative images of DAPI-stained nuclei fixed at successive time points post-ablation. Nuclei in the ablated region (dashed line), become progressively more irregular and then condense as they undergo pyknosis (arrowheads). (E) Representative images of embryos fixed at successive time points post-ablation with cell membranes marked by β-catenin. Cell membrane integrity is initially disrupted in the ablated region and gradually heals over time. 0 h: ablated, *n*=3; control, *n*=4. 1 h: ablated, *n*=5; control, *n*=5. 2 h: ablated, *n*=7; control, *n*=6. (F) Apoptotic cells marked by activated Caspase 3 are seen after 1 h in the region of ablation. By 2 h post-ablation, remaining apoptotic cells and debris are localised laterally as the apoptotic debris flows out of the tailbud. 1 h: ablated, *n*=4; control, *n*=4. 2 h: ablated, *n*=6; control, *n*=6.

Fate mapping in the zebrafish tailbud has so far shown that dorsal progenitors are a majority neural-fated population whereas ventral and lateral progenitors are mesodermal-fated (Fig. 1A). At the single-cell level, there is unlikely to be a clear boundary, but fate-mapping studies show that there does not appear to be extensive mixing between the two domains (Attardi et al., 2018; Kanki and Ho, 1997; Lange et al., 2024; Lawton et al., 2013). NMC cells are often associated with the co-expression of the transcription factors *sox2* and *tbxta* (Binagui-Casas et al., 2021) and, in zebrafish, cells with this gene expression state are located in the posterior wall of the tailbud (Martin and Kimelman, 2012; Attardi et al., 2018). This region spans both dorsal and ventral progenitors and, importantly, is heterogeneous, with *sox2^+^/tbxta^+^* cells mixed with *sox2^+^* (dorsally) and *tbxta^+^* (ventrally) (Fig. 1A) (Toh et al., 2022). For the purposes of our study, we took a morphological approach to classifying tailbud progenitors by splitting them into either dorsal (and therefore likely to be mostly neural-fated) or ventral (and therefore likely to be mostly mesodermal-fated) relative to the notochord progenitors.

We first set out to ablate the dorsal progenitors in the tailbud at the 14-somite stage, to determine whether this affected the proportional formation of spinal cord relative to paraxial mesoderm. The region of interest (ROI) was localised dorsally and posteriorly to the morphologically distinguishable notochord progenitors. This region was clearly within the *sox2*-positive region of the tailbud and was expected to include both *sox2^+^* and *sox2^+^/tbxta^+^* dorsal progenitors (Fig. 1B). Mapping ablations together showed that there was a high precision in user-selected ablation ROIs (Fig. 1C), meaning that we were reliably targeting dorsal progenitor cells.

Next, to validate that two-photon ablation causes sufficient and rapid cell death under our experimental conditions, we examined fixed samples at intervals following ablation. In these samples, we observed irregular nuclear staining (Fig. 1D) and the destruction of cell membranes (Fig. 1E) immediately following ablation. This progressed to obvious nuclear pyknosis (Fig. 1F) at 1-2 h post-ablation as the ablation decreased in size (Fig. 1E). At this stage, staining for activated Caspase 3 showed that cells in the dorsal-posterior region were undergoing apoptosis. Apoptotic debris moved rapidly through the tailbud and was subsequently observed in the lateral region of the tailbud following the flow of cells (Fig. 1F; Movie 1).

**Fig. 2.**
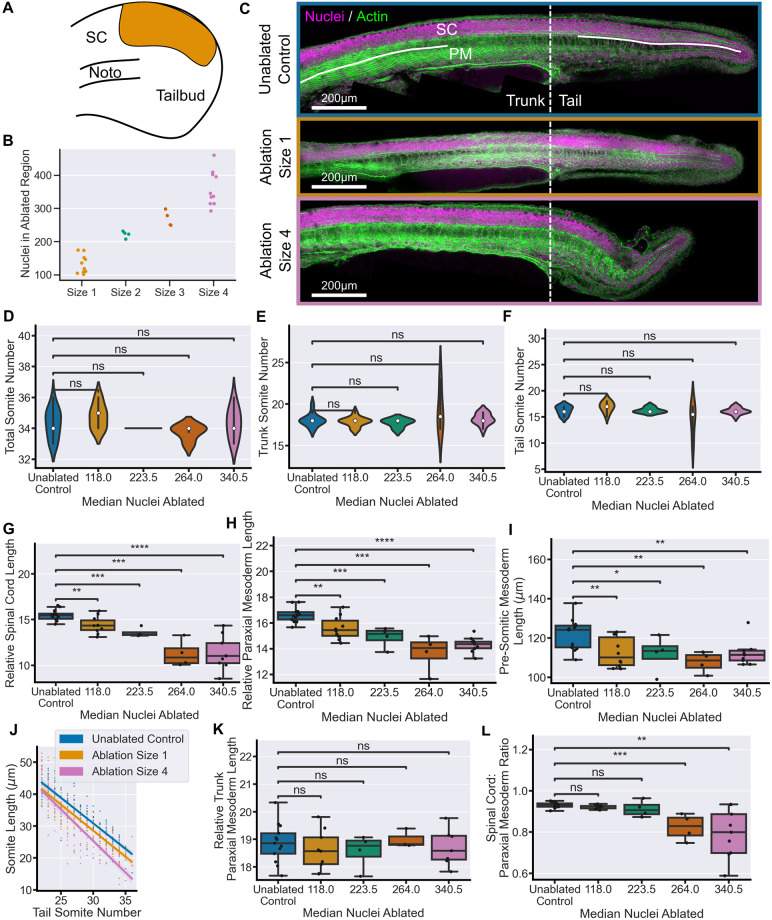
**Dorsal progenitor ablation results in a proportional reduction of tail tissue elongation.** (A) Schematic showing an example of dorsal progenitor ablation (orange area) in the 14-somite stage tailbud. (B) Number of nuclei in the ablation ROI prior to ablation at different ROI sizes. (C) Representative examples of embryos at 30 hpf stained for nuclei and actin. The morphology of the tail is comparable between size 1 ablations and unablated controls, whereas size 4 ablations cause a clear defect in tail formation. Solid lines indicate the regions measured in G and K. (D-F) Total somite counts (D), as well as trunk (E), tail somite number (F), are comparable between control embryos and all ablation conditions. (G,H) Spinal cord length (G) and paraxial mesoderm length (H), measured from the 22nd somite, relative to total somite number, both show a significant decrease in all ablated conditions compared to controls. (I) Pre-somitic mesoderm length shows a significant decrease in all ablated conditions compared to controls. (J) Average somite length is consistently decreased in size 1 ablation compared to controls. In size 4 ablations, there is a more notable decrease in the most posterior somites. (K) Trunk paraxial mesoderm length (2nd to 10th somites), relative to total somite number, shows no significant difference between any ablated condition and controls. (L) Spinal cord length relative to mesoderm length from the 22nd somite shows that ablations of size 1 and 2 maintain a ratio of tail tissues comparable to control embryos while size 3 and size 4 embryos have a significantly lower ratio. Unablated control, *n*=13; size 1, *n*=10; size 2, *n*=4; size 3, *n*=4; size 4, *n*=9. Conditions were compared using Mann–Whitney-Wilcoxon test. *P≤0.05; **P≤0.01; ***P≤0.001; ****P≤0.0001. Noto, notochord; ns, not significant; PM, paraxial mesoderm; SC, spinal cord. Relative length has units of µm/somite.

We then ablated dorsal progenitors (Fig. 2A) in groups of embryos using ROIs of increasing size, from which we quantified the number of nuclei in the ablated region as an estimate of the number of cells removed from the tailbud (Fig. 2B; Fig. S1A). These embryos were left to grow, alongside unablated control embryos, until the end of somitogenesis 30 h post-fertilisation (hpf)] when they were fixed and stained for nuclei and actin to allow morphological identification of all tail tissues. Qualitatively, ablations of size 1 (median 118 nuclei) and 2 (median 223 nuclei) had little effect on the morphology of any of the tail tissues (Fig. 2C; Fig. S1B). We estimate, based on quantification of the number of *sox2*^+^ nuclei in the tailbud at this stage, that these ablations accounted for 9-17% of *sox2*^+^ cells. Ablation of a larger proportion of dorsal progenitors, size 3 (median 264 nuclei) and 4 (median 340 nuclei) (Fig. 2B; Fig. S1A), did cause a reduction in tail spinal cord size and overall tail elongation (Fig. 2C; Fig. S1B). We quantified the number of somites to determine whether ablation affects their production and found that, with one exception in the size 3 ablations, the ablation conditions did not alter the total number of somites (Fig. 2D), or the number within either the trunk or the tail (Fig. 2E,F), as measured from the yolk extension. From this, we can conclude that ablations do not affect the somitogenesis clock.

To quantify whether ablation affects the elongation of the spinal cord and/or paraxial mesoderm, we measured the length of both tissues in the tail, beginning at the 22nd somite, which is always located in the tail (Attardi et al., 2018). The spinal cord was measured to the tip of the spinal cord lumen, and the paraxial mesoderm was measured to the tip of the tail. The length of each tissue was normalised to the number of somites to account for slight variation in developmental timing amongst embryos by dividing by total somite number. We found that even the smallest ablations caused a significant decrease in the elongation of the tail spinal cord, with the effect even more pronounced following the largest ablations (Fig. 2G). This demonstrates that dorsal progenitors in the tailbud are required for spinal cord elongation in the posterior body. Importantly, the loss of dorsal progenitors also significantly affected the elongation of the tail paraxial mesoderm (Fig. 2H). This decrease in elongation was consistent in the PSM (Fig. 2I) as well as in the somites across the tail (Fig. 2J), but could not be seen in the length of trunk somites, which are comparable between conditions (Fig. 2K).

Comparison of the lengths of the two tissues relative to one another showed that the spinal cord and paraxial mesoderm lengths scale their elongation following ablations of size 1 (median 118 nuclei) and size 2 (median 223 nuclei). There is a limit to this ability as proportional reduction breaks down following larger ablations, around 264 nuclei or more. In these cases, the elongation of the spinal cord was more affected than the paraxial mesoderm, likely due to the large-scale loss of dorsally located neural progenitors (Fig. 2L). Taken together, these data demonstrate that the proportional extension of neural and mesodermal tissue in the tail has a considerable capacity for regulation following the loss of dorsal progenitors.

### *sox2* and *tbxta* gene expression is robust to loss of dorsal progenitors

**Fig. 3.**
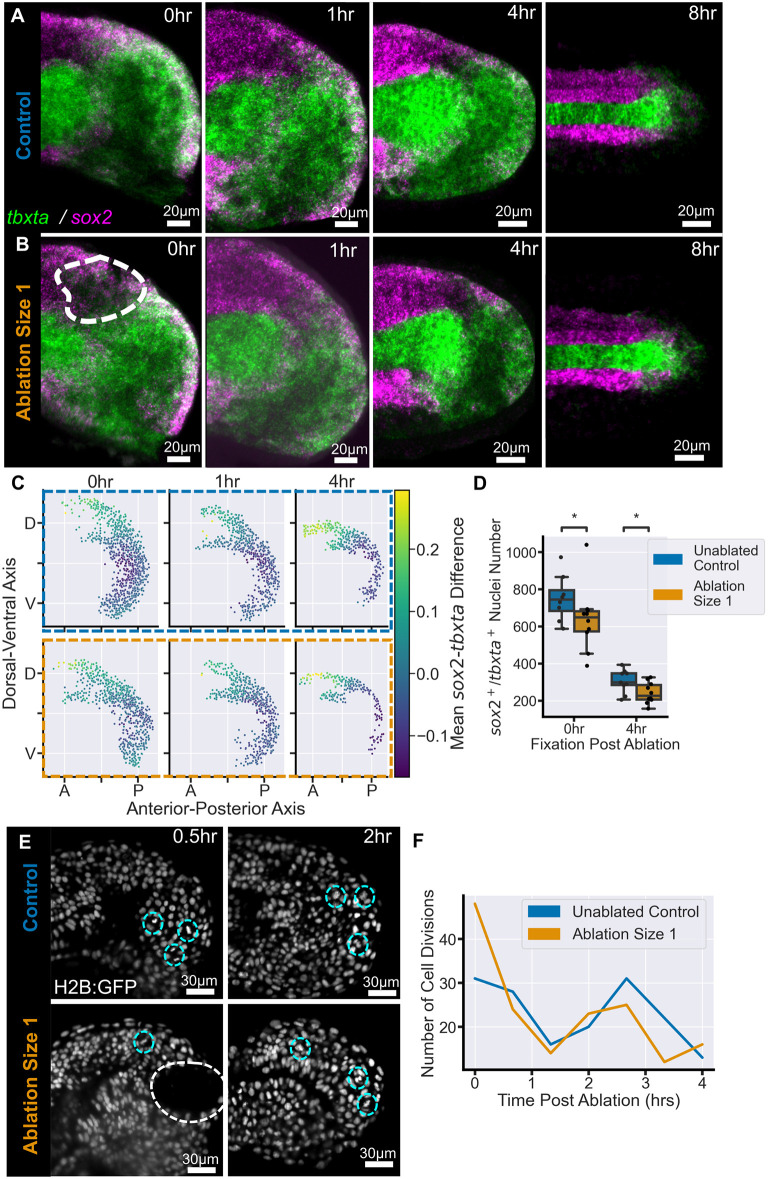
**Dorsal progenitor ablation does not affect the gene expression pattern or cell division levels in the tailbud.** (A,B) Mean (average) projection through the midline of representative images of the *sox2/tbxta* expression pattern in control (A) and ablated (B), embryos over time. (B) Initial disruption of the pattern can be seen in ablated embryos at 0 h post-ablation (white dashed outline). By 4 h, and up to 8 h, post-ablation the gene expression pattern is comparable to control embryos. (C) Average map of the *sox2^+^/tbxta^+^* cells from tailbuds at each time point in control and ablated embryos. The axes show the distribution of these cells in the dorsal-ventral and anterior-posterior axes and the average level of *sox2* versus *tbxta* expression within 11 µm of each point. There is no significant change in the average maps between control and ablated embryos. (D) Number of *sox2/tbxta* double-positive nuclei in each tailbud. Ablation causes a significant drop in the number of nuclei at 0 h post-ablation compared to controls. A significant reduction remains at 4 h post-ablation compared to controls. 0 h: ablated, *n*=10; control, *n*=8. 1 h: ablated, *n*=11, control, *n*=9. 4 h: ablated, *n*=10; control, *n*=9. 8 h: ablated, *n*=3; control, *n*=3. (E) Long-term lightsheet imaging of an ablated embryo (white dashed circle) and a stage-matched control allows manual identification of dividing cells (cyan dashed circles). (F) There is no clear difference between the number of divisions in ablated and control embryos over 4 h. Ablated, *n*=1; control, *n*=1. Conditions were compared using Mann–Whitney-Wilcoxon test. *P≤0.05.

NMC cells are a potential candidate for facilitating the regulation of neural and mesodermal proportions as they could balance the relative number of neural versus mesodermal fated progenitors. To investigate whether this is the case, we fixed embryos at discrete intervals following size 1 ablations (on average 118 dorsal nuclei) and stained them for the expression of the transcription factors *sox2* and *tbxta* (Fig. 3A,B). These genes are involved in the early steps of neural (Okuda et al., 2010) and mesodermal (Martin and Kimelman, 2008, 2010) differentiation, respectively, and their co-expression is commonly associated with a cell in an NMC state (Wymeersch et al., 2021; Binagui-Casas et al., 2021). In zebrafish, the *sox2*^+^*/tbxta*^+^ cells, which correspond to the NMC progenitors, are located in the posterior wall of the tailbud (Martin and Kimelman, 2012; Attardi et al., 2018; Toh et al., 2022).

Examination of the time course of *sox2* and *tbxta* expression following ablation showed that there is an initial decrease in expression in the location of the ablation immediately after it has occurred (compare left-hand panels in Fig. 3A,B; dashed outline). One-hour post-ablation, some disorganisation of the gene expression pattern remained, but by 4 h post-ablation (Fig. 3B) ablated embryos were qualitatively similar to control embryos (Fig. 3A). Importantly, *sox2/tbxta* co-expressing nuclei were still located in the dorsal and posterior wall of the tailbud. At the end of somitogenesis (8 h post-ablation), the *sox2*^+^*/tbxta*^+^ cells had differentiated and a *sox2*^+^ spinal cord could be seen in both control and ablated embryos, whereas the tailbud remnants expressed *tbxta* (Fig. 3A,B).

There was variation in the expression pattern both within and between control and ablated embryos, for example in the dorsal extent of *tbxta* (Fig. S2A, dashed line); this is something we have previously demonstrated is characteristic of NMC cell gene expression in wild-type embryos (Toh et al., 2022). Therefore, to understand whether the general pattern of *sox2/tbxta* expression is meaningfully affected we segmented individual nuclei within the tailbud in three dimensions and quantified the mean intensity of each gene within each nucleus. Each nucleus was represented as a single point and coloured according to whether it expressed *sox2* and/or *tbxta* (Fig. S2B). For *sox2/tbxta* co-expressing nuclei, we calculated the relative levels of each gene as a measure of their NMC state (Fig. 3C; Fig. S2C) and, finally, we registered the embryos together to average the gene expression of nearby nuclei across replicates (Fig. 3C). In both control and ablated embryos, we found that at early stages the NMC domain is broad and the nuclei have similar levels of *sox2* and *tbxta*, but as development continues the domain thins and some nuclei have higher levels of *sox2* or *tbxta* (Fig. 3C; Fig. S2C). This indicates that ablation does not significantly impede this stage of NMC differentiation and the progression of the change in the NMC state. There does appear to be a slight change to the extremes of the data; there was a decrease in the number of *sox2*-high cells and a slight over-representation of *tbxta*-high cells, 4 h after ablation compared to the 95th percentile of the control data (Fig. S2C). However, this did not translate to a change in the average distribution of *sox2/tbxta* levels mapped onto their location in the tailbud, which showed a high degree of similarity between control and ablated embryos at all stages (Fig. 3C). Even the larger size 2 ablations, which resulted in a tailbud that was clearly depleted in *sox2* and *tbxta* co-expression, maintained some expression in the posterior wall (Fig. S2D, arrowheads), although analysis was hampered by spikes of expression associated with dying cells in the spinal cord (Fig. S2D, asterisk).

To investigate this further, we performed ablations in Sox2::GFP and Tbx16::GFP transgenic embryos, then fixed and stained the embryos after 2 h for GFP to detect the activation of the transgenes. At this stage, we observed that the ablation had little impact on the distribution of Sox2::GFP signal, although as noted above there was some variability (Fig. S2E). Tbx16 expression marks a cell's initial commitment to mesoderm identity (Kinney et al., 2020) so any change to the localisation of Tbx16::GFP fluorescence would imply that ablation can seriously affect cell fate patterning. However, we found that Tbx16::GFP remains localised to the PSM and the endothelial mesoderm with no evidence of any shift into the dorsal region caused by the ablation (Fig. S2F and Fig. S3D). Taken together, these results show that the spatial localisation and temporal changes of the *sox2* and *tbxta* (NMC) expression patterns in the tailbud are robust to dorsal progenitor ablation.

### NMC cells do not increase division levels in response to ablation

The robustness of the NMC gene expression pattern could mean that NMC cells balance out their neural and mesodermal contribution to regulate tail length; however, for this to occur there would have to be either an increase in NMC number to replace the nuclei lost by ablation or a shift in the movement of the cells to repopulate the lost dorsal progenitors at the expense of ventral mesodermal progenitors.

To investigate the first hypothesis, we quantified the number of *sox2*^+^*/tbxta*^+^ cells in each tailbud to determine whether there is an increase associated with higher division levels. Instead, we found that immediately following ablation there is a significant drop in the number of NMC cells (Fig. 3D), as expected from the loss of gene expression in the dorsal-posterior tailbud wall (Fig. 3B); however, this decrease was not recovered over time and remained significant after 4 h post-ablation (Fig. 3D). Additionally, we counted the nuclei in the posterior-most tip of the spinal cord at the end of somitogenesis (8 h post-ablation) to determine whether there is a later replacement of the lost cells in the spinal cord. Although not statistically significant, we did find that two out of three embryos had fewer nuclei and a reduction in spinal cord height in comparison to control embryos (Fig. S3A-C). In contrast to the drop in NMC nuclei number, the number of *tbx16*^+^ nuclei was not decreased at either time point in ablated embryos (Fig. S3C,D).

To further verify a lack of cell division, we quantified the number of divisions occurring in the tailbud over the course of 4 h following ablation (Fig. 3E) and found no difference compared to the number of divisions in the unablated tailbud (Fig. 3F). Together, these results indicate that neural progenitor ablation does not trigger an increase in cell division to replace the lost cells in the tailbud.

### NMC cells do not shift their migration following ablation

Alternatively, NMC cells could be changing their position in the tailbud to repopulate the dorsal progenitors at the expense of mesodermal differentiation. In the zebrafish tailbud, the localisation of the NMC cells is linked to their pattern of differentiation with the dorsally located cells becoming neural and the ventrally located cells becoming mesodermal (Attardi et al., 2018; Toh et al., 2022). Therefore, in order to balance the loss of dorsal progenitor cells we would expect to see a shift from the ventral mesodermal-fated region towards the dorsal neural-fated region, potentially as the result of cell rearrangements during ablation healing.

**Fig. 4.**
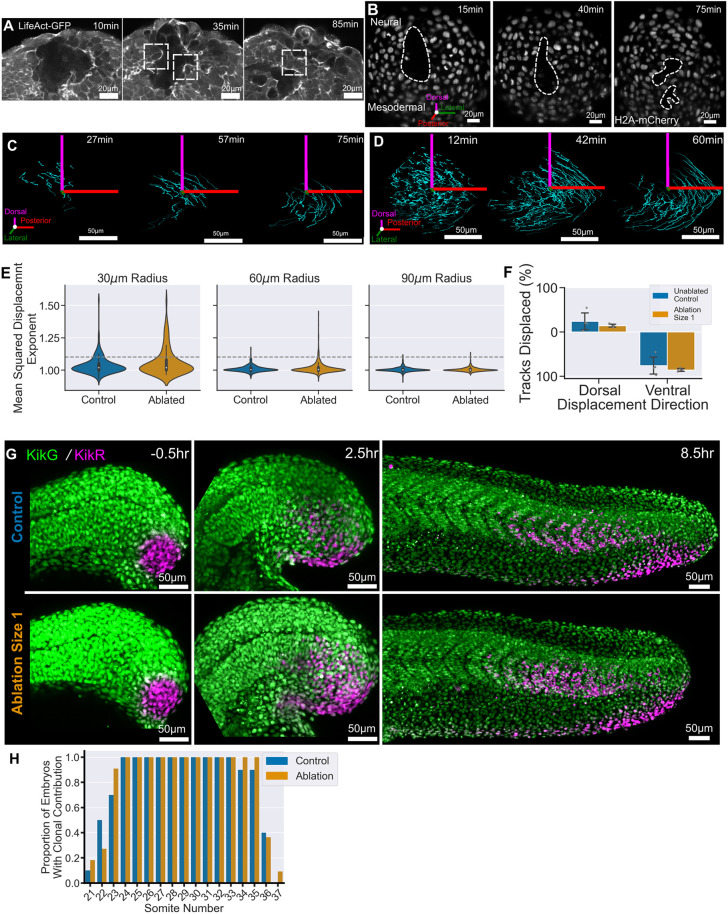
**Dorsal progenitor ablation does not perturb global cell movements.** (A) Representative images of ablation healing visualised using Lifeact:GFP. Cells increase actin levels at the at the ablation edge and move into the ablated region to re-establish cell contacts (boxes). (B) Representative images of ablation healing using H2A:mCherry to mark nuclei; these nuclei were identified and tracked over 1 h. Ablated, *n*=4; control, *n*=4. (C,D) Cell tracks in a single embryo (in this case an ablated embryo) can be split into more consistent (C) and less consistent (D) movement. (E) The exponent of the mean squared displacement for each track across all embryos gives a measure of the consistency of motion. Tracks are grouped according to their starting distance relative to the ablation centre or equivalent control point. There are a greater number of tracks with a more consistent motion in ablated embryos, in particular close to the ablation, compared with control embryos (above the grey dashed line). (F) The displacement of each track in the dorsal-ventral axis relative to its starting position. The vast majority of tracks are displaced in the mesodermal direction in both control and ablated embryos. Ablated, *n*=4; control, *n*=4. (G) Representative examples of tracing the fate of a group of ventral and lateral progenitor cells using the photoconvertible Kikume protein in control and ablated embryos. Ventral progenitors were photoconverted and half were subsequently ablated before being grown to the end of somitogenesis. In both control and ablated embryos, there is no movement of the ventral progenitors into the spinal cord. Control: *n*=10; ablated *n*=11. (H) The proportion of embryos shown in G with some contribution of labelled cells to each posterior somite was calculated. Both control and ablated embryos show similar ventral progenitor contribution to the posterior somites.

To explore whether this process occurs, we live imaged embryos following ablation. Observation of LifeAct-GFP and MyosinII-mCherry transgenic lines allowed visualisation of the ablation healing process occurring over 1.5 h (Fig. 4A; Fig. S4B). Of particular importance, are the cells on the edge of ablation that moved into the ablated region and formed new cell–cell contacts (Fig. 4A, boxed areas). This healing process was also associated with increased activity of both Actin and non-muscle Myosin II at the edge of the cells exposed to the ablation, quintessential characteristics of a wound-healing process (Fig. 4A; Fig. 4B, Movie 2). These features are suggestive of changes to local cell behaviour caused by ablation.

To examine local changes to behaviour in greater detail, we live imaged nuclei (Fig. 4B) and extracted tracking information describing their individual movements over time, in three dimensions (Fig. 4C,D). We then selected only those tracks which began in the first 10 min of the movie and calculated their distance from the centre of the ablation or a point in an equivalent location of an unablated control embryo. A common metric for quantifying cell motion on a spectrum of diffusive to directional is the mean squared displacement (MSD). The exponent of the MSD curve gives the degree of directional motion at values greater than 1.0 (Lawton et al., 2013; Beltman et al., 2009; Hu et al., 2023). Plotting the distribution of this value for all tracks at different distances from the ablation centre (or equivalent in control embryos) showed that the majority of tracks had an MSD exponent of around 1.0, which is indicative of random migration (Fig. 4E). A user-defined threshold was set at an exponent of 1.1 to select tracks that had less random, and therefore more directional, motion (grey dashed line). We observed that both ablated and control embryos contain a subset of tracks that have a relatively high MSD exponent (Fig. 4C). Importantly, there was a greater proportion of these tracks in ablated embryos and particularly among tracks that began close to the ablation (Fig. 4C,E). These tracks also exhibited other measures of directional motion, such as a high corrected straightness (Fig. S4B), and a high mean angular displacement (Fig. S4C). An example of a nucleus that produced one of these directional tracks is shown in Fig. S4D; here, the cell is protruding into the ablated region and forming a new cell–cell contact (arrowhead). We did not observe any consistent decrease in velocity that might be associated with these tracks or localised in some way within the tailbud (Fig. S4E). Taken together, these observations suggest that ablation does cause a small, local increase in the amount of directional motion.

Although ablation healing does alter the behaviour of a subset of nuclei, we noticed that, even as it is healing, the ablation is displaced ventrally (Fig. 4B; Movie 3). Similarly, the tracks, both those that display more directional movement (Fig. 4C) and those that do not (Fig. 4D), all displayed a clear dorsal-to-ventral flow over the course of the movie. This would suggest that the ablation is not sufficient to cause a ventral-to-dorsal shift in the distribution of progenitors in the tailbud. To confirm this, we calculated the percentage of tracks for each embryo within 60 µm of the ablation that are displaced in either the dorsal or ventral direction. We found that the majority of tracks in each embryo were displaced towards the ventral region in both control and ablated embryos (Fig. 4F).

Despite the lack of a ventral-to-dorsal shift during ablation healing, there could be a later shift in the tailbud. To investigate this, we carried out a labelling experiment using the photoconvertible Kikume protein. A region of ventral progenitors was labelled in a group of embryos and the dorsal progenitors were ablated in half of the embryos. If there is a later shift in the fate of NMC cells, we would expect to see labelled progenitors entering the neural tube towards the end of somitogenesis. However, in all embryos, both control and ablated, we observed that the ventral progenitors contribute to only mesodermal structures and not neural ones (Fig. 4G). Importantly, we counted the proportion of embryos that displayed some clonal contribution to the posterior somites and found that this was also comparable between control and ablated embryos, suggesting that the ablation is not affecting the extent of mesoderm contribution (Fig. 4H). This finding correlates with a long-term live movie from a large ablation in which the remaining debris can be seen to move as a clump and flow into the mesodermal rather than neural domain (Fig. S4F). Consequently, we conclude that there is not a significant shift in the ventral progenitors to compensate for the reduction in dorsal progenitors.

We also investigated whether there is a change to Wnt signalling in the tailbud following dorsal progenitor ablation. Wnt is the key signal in the neuromesodermal fate decision: high levels of Wnt are associated with mesoderm differentiation and low levels are associated with neural differentiation (Martin and Kimelman, 2012). Under this model, NMC cells have an intermediate level of Wnt (Bouldin et al., 2015) and the Wnt environment is self-sustaining through a *tbxta* positive feedback loop (Martin and Kimelman, 2008). Consequently, we would expect that the Wnt environment scales to the number of *tbxta*-expressing progenitors and is therefore robust to changes in cell number. However, if there is a shift in the distribution of progenitors from ventral to dorsal regions in the tailbud we might expect to see higher levels of Wnt signal in the dorsal region after ablation.

**Fig. 5.**
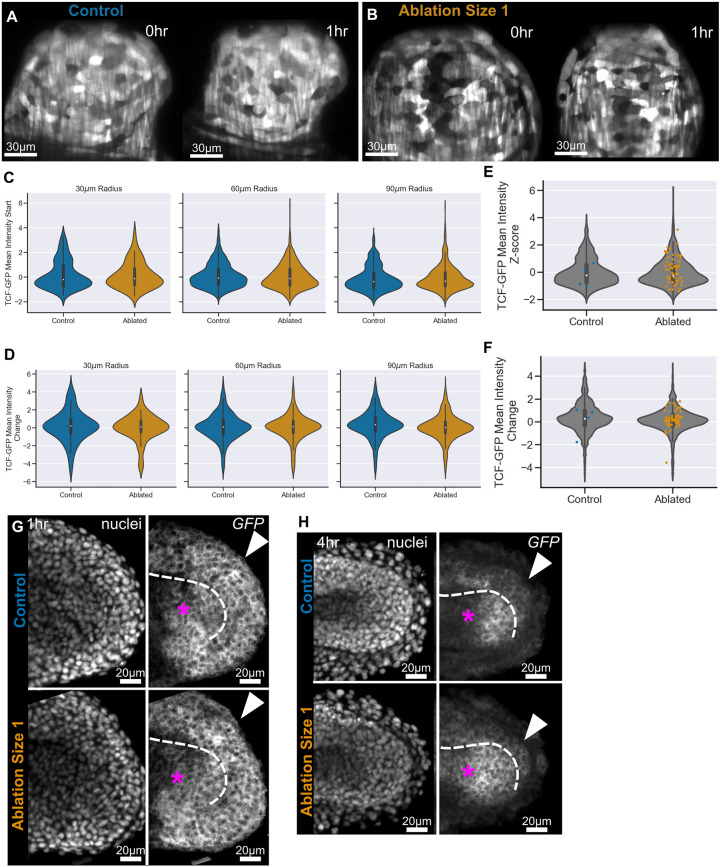
**Global cell flow and Wnt signalling transduction are robust to dorsal progenitor ablation.** (A,B) Representative images of TCF-GFP highlighting the level of Wnt signalling transduction in control (A) and ablated (B) embryos. (C) TCF-GFP intensity, z-score normalised for each embryo, at the start of each track for tracks at different distances from the ablation/reference point. Tracks close to the ablation have comparable levels of TCF-GFP to control embryos. (D) Mean TCF-GFP intensity change over the track show both increase and decrease in Wnt transduction with ablated embryos having a similar distribution to control embryos. (E,F) TCF-GFP starting intensity (E) and TCF-GFP mean intensity change (F) for all tracks within 60 µm of the ablation (grey) overlaid with the intensity of the directional tracks (coloured) shows that directional tracks do not have a bias in Wnt transduction. Ablated, *n*=2; control, *n*=2. (G,H) Mean (average) projections through the midline of representative images of control and ablated TCF-GFP embryos at 1 h (G) and 4 h (H) post-ablation. Embryos were stained for *GFP* transcript to pick up any rapid changes to transcription from the TCF-GFP transgene. (G) Transduction of Wnt signalling through the transgene is widespread in the posterior wall (arrowheads) and notochord progenitors (asterisks) in both control and ablated embryos at 1 h post-ablation. (H) Transduction of Wnt signalling through the transgene is comparable between control and ablated embryos in the posterior wall (arrowheads) but appears to be elevated in the notochord progenitors (asterisks).

To investigate this, we imaged embryos with a *Tg(7xTCF-Xla.Sia:GFP)* transgene (hereafter referred to as TCF-GFP) to visualise the transduction of Wnt signalling. This reporter line has seven TCF-binding sites upstream of the *Xenopus siamois* promoter driving the production of GFP, and the transgene is active in response to the transduction of Wnt signalling and marks key Wnt domains in the embryo (Moro et al., 2012). We observed that there is great variation in the level of Wnt transduction as reported by this transgene in both control and ablated embryos (Fig. 5A,B). This was confirmed by the broad distribution of normalised mean intensity values at the start of each track (Fig. 5C). We then calculated the mean change in intensity across the track lifetime and found that the vast majority of tracks did not change their level of TCF-GFP in either control or ablated embryos, with a minority of tracks in both conditions either increasing or decreasing their Wnt transduction (Fig. 5D). Additionally, more directional cell motion did not correlate with a particular level of Wnt signalling (Fig. 5E) or a significant change in the level of Wnt transduction (Fig. 5F).

It is possible that the ablation healing is too rapid to observe a change in levels of GFP signal. Moro et al. (2012) found that changes to Wnt signalling were detected at the transcript level within 3 h, so we examined mRNA for GFP at 1 h and 4 h after ablation. At 1 h post-ablation, *GFP* was widespread within the tailbud but showed little difference between control and ablated embryos, particularly in the dorsal posterior wall (Fig. 5G, arrowheads). By 4 h post-ablation, *GFP* transcript in the dorsal posterior region remained comparable between ablated and control embryos (Fig. 5H, arrowheads), although there appeared to be an expansion of expression in the notochord progenitors (Fig. 5H, asterisks). These data do not demonstrate any clear increase in levels of Wnt transduction in the dorsal region of the tailbud, suggesting that mesodermal-biased progenitors are not moving into the dorsal region. Further investigation will be required to determine whether the increase in Wnt transduction in the notochord is a direct consequence of ablation or an indirect one, perhaps caused by a reduction in tail elongation.

Taken together, these data demonstrate that the cells that heal the ablated region are the neighbouring, dorsal cells rather than more distantly located ventral cells. Without a shift in cell localisation, it is unlikely that NMC cells would be able to alter their differentiation ratio. Therefore, we conclude that NMC cells do not play a significant role, following dorsal ablation, in the regulation of neural and mesodermal tissue proportions.

### Tail elongation can withstand the loss of mesoderm progenitor cells

Our results so far demonstrate that dorsal progenitor ablation does not cause a change in gene expression distribution, cell division, or cell movement in the tailbud. This provides considerable evidence against the hypothesis that NMC cells play a key role in proportional regulation of spinal cord and paraxial mesoderm length. In the absence of increased divisions, the lost cells are not replaced so this hypothesis hinges on the possibility that the population of NMC cells adjusts its relative contribution to neural versus mesodermal differentiation, i.e. some mesoderm-fated NMC cells instead contribute to spinal cord, thus causing a decrease in paraxial mesoderm length. Our observations that cell movements are not affected in both the short and the long term suggests that there is no re-distribution of NMC cells. We set out to perform a final experiment that challenges the NMC cell rearrangement hypothesis, i.e. to determine whether a direct reduction in mesoderm progenitor number causes a similar decrease in tail elongation as an ablation of the dorsal progenitors (Fig. 2). If NMC cell re-distribution is causing the proportional reduction in tissue elongation, then we would expect that a direct ablation of mesoderm progenitors would cause a similar phenotype.

**Fig. 6.**
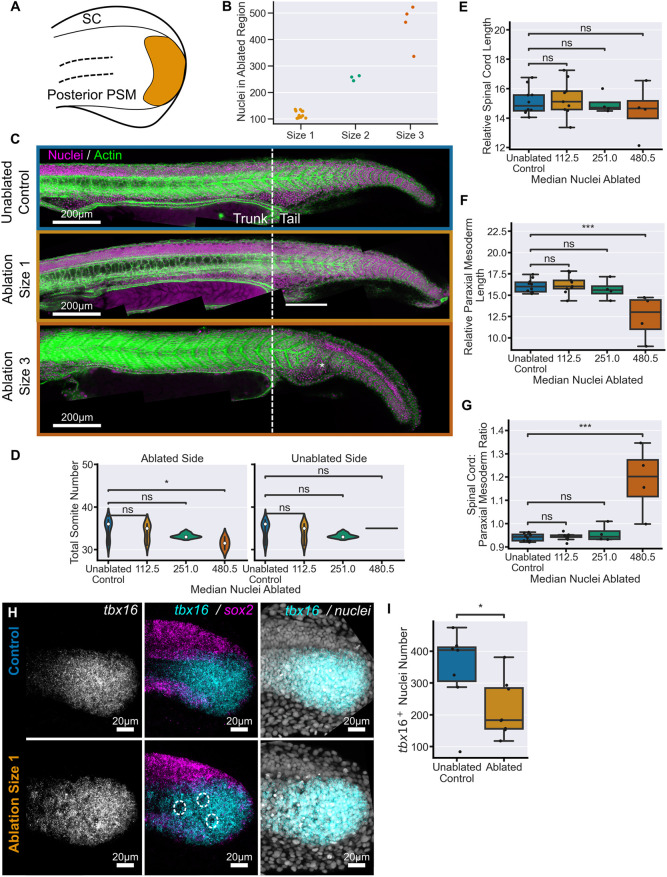
**Direct removal of mesoderm progenitors does not affect tissue elongation.** (A) Schematic showing the location of an example mesodermal-fated lateral progenitor ablation (orange area) in the 14-somite-stage tailbud. (B) Number of nuclei in the ablation ROI prior to ablation at different ROI sizes. (C) Representative examples of embryos at 30 hpf stained for nuclei and actin. The morphology of the tail is comparable between size 1 ablations and unablated controls, whereas size 3 ablations cause a clear defect in somite formation (asterisk). (D) Total somite count for both sides of the bilateral paraxial mesoderm. Size 1 and size 2 ablations have comparable numbers of somites on both sides to control embryos. In size 3 ablations, there is a loss of somites on the ablated side but the contralateral side remains unaffected compared to controls. (E,F) Spinal cord length (E) and paraxial mesoderm length (F), measured from 22nd somite, relative to total somite number, both show no significant decrease in size 1 and 2 ablations compared to controls. Size 3 ablations do have a significant difference in paraxial mesoderm length only. (G) Spinal cord length relative to mesoderm length from the 22nd somite shows that ablations of size 1 and 2 maintain a ratio of tail tissues comparable to control embryos while size 3 embryos have a significantly higher ratio. Control, *n*=10, size 1, *n*= 9; size 2, *n*=4; size 3, *n*=4. (H) Lateral maximum projections of representative embryos stained for *sox2* and *tbx16* mRNA from control and mesoderm progenitor ablations at 4 h post-ablation. The *tbx16* domain is comparable between control and ablated embryos; however, the cell debris creates noticeable holes in the pattern (dashed circles). (I) Nuclei from the control and ablated tailbuds shown in H were segmented in three dimensions and the number of *tbx16^+^* nuclei were counted. At 4 h post-ablation, there is still a significant decrease in *tbx16* nuclear number in ablated embryos compared to controls. Control, *n*=7; ablated, *n*=8. Conditions were compared using Mann–Whitney-Wilcoxon test. *P≤0.05; ***P≤0.001. ns, not significant; PSM, pre-somitic mesoderm; SC, spinal cord. Relative length has units of µm/somite.

We ablated paraxial mesoderm progenitors on one lateral side of the tailbud (Fig. 6A). As with dorsal progenitor ablations, we performed ablations of different sizes – designated 1 to 3 (Fig. 6B; Fig. S5A) – and grew the embryos until the end of somitogenesis (Fig. 6C; Fig. S5B). Importantly, these ablation sizes were comparable to those performed in the neural progenitor region (Fig. 2B; Fig. 6B). Similar to neural progenitor ablations, there were no morphological defects at the initial ablation sizes 1 and 2 (median 112 and 251 nuclei, respectively). Size 1 ablations counted for 14% of the average number of *tbxta*^+^ nuclei in the tailbud at this stage. At the largest ablation size, several somites were lost from the tail on the side of ablation (Fig. 6C,D, asterisk). Notably, quantification of somites on the unablated side showed that large mesodermal ablations only caused the loss of somites on the ablated side and did not appear to disrupt the somitogenesis clock (Fig. 6D).

In contrast to dorsal progenitor ablation, there was no reduction of spinal cord (Fig. 6E) or paraxial mesoderm (Fig. 6F) length in the tail following mesodermal progenitor ablations of up to 250 cells. Additionally, we observed no difference in length between the ablated and unablated sides of the paraxial mesoderm (Fig. S5C). This provides clear evidence that a re-distribution of NMC cells within the tailbud following neural progenitor ablation cannot account for the proportional decrease in paraxial mesoderm length. Importantly, fixing embryos 4 h after ablation and staining for *tbx16* showed similar expression patterns between control and ablated embryos, although holes associated with nuclear debris could be seen throughout the region (Fig. 6H). This is in line with the documented fluidity of this tissue due to extensive cell rearrangements (Lawton et al., 2013; Mongera et al., 2018; Thomson et al., 2021). Quantification of the number of *tbx16*^+^ nuclei showed that the ablated cells were not replaced by this stage (Fig. 6I). However, by the end of somitogenesis PSM length and nuclei number were comparable between control and ablated embryos (Fig. S5D,E), suggesting either a replacement of cells or a re-distribution of mesodermal progenitors such that the effects of the ablation are distributed throughout the paraxial mesoderm.

A reduction in paraxial mesoderm elongation was only seen following the largest ablations of around 480 cells where it caused a deregulation of tissue proportions, as spinal cord elongation was not affected (Fig. 6G). Interestingly, this result suggests that even when paraxial mesoderm elongation is decreased it has less of an effect on spinal cord elongation than the reverse scenario (Fig. 2). Taken together, we conclude that tail elongation is particularly sensitive to the loss of dorsal progenitors, a result that is not consistent with the hypothesis of NMC cell re-distribution.

### Ablation of spinal cord cells causes a reduction in neural and mesodermal tissue length

In the absence of changes to NMC cell behaviour following dorsal progenitor ablation, we turned to investigate our alternative hypothesis, that spinal cord morphogenesis drives tail elongation and consequently tissue proportions. In this scenario, dorsal progenitors are important not because they include *sox2*^+^*/tbxta*^+^ cells but because they are majority fated to become spinal cord. If this is the case, then a reduction in spinal cord cell number anterior to the tailbud should also affect the length of the tail tissues.

Ablation of dorsal neural progenitors is likely to have a large knock-on effect as a whole progenitor lineage is lost for each cell ablated. Therefore, to reduce a large number of cells along the whole spinal cord we turned to a genetic ablation technique for tissue-specific expression of the bacterial Kid toxin (Labbaf et al., 2022). The expression of UAS:Kid in the spinal cord was driven by an Adamts3:GAL4FF (ATS3:GAL4) transgene, which has strong expression in motor neurons (Asakawa et al., 2013; Wang et al., 2020). Our ATS3:GAL4 line also contains a UAS:GFP cassette to visualise activity of ATS3.

**Fig. 7.**
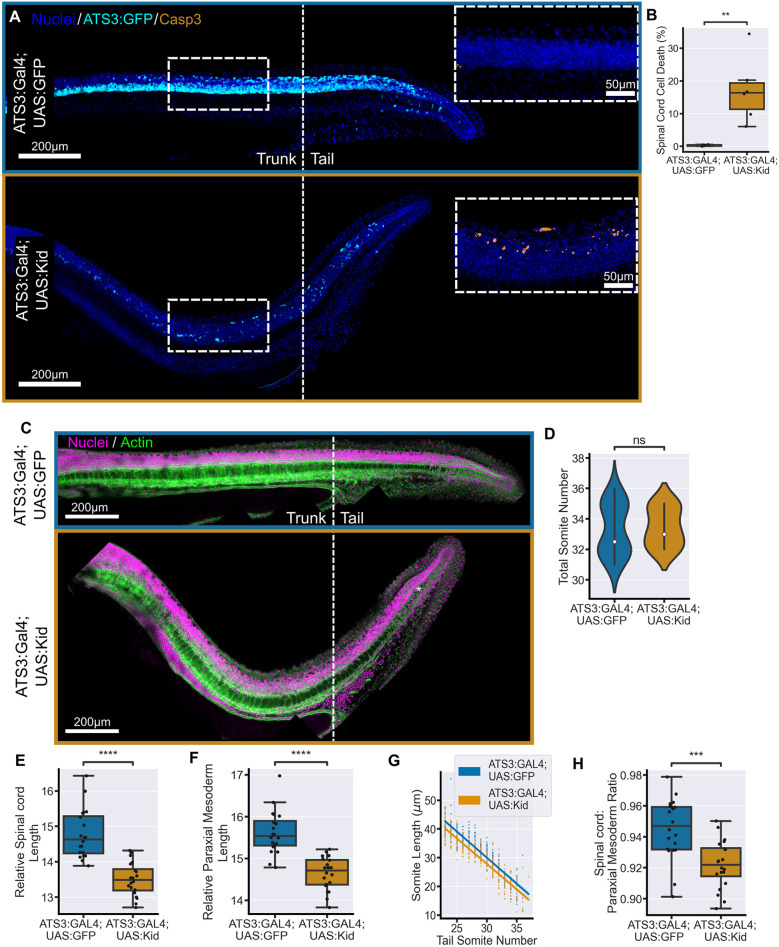
**Genetic ablation of spinal cord cells along the body axis results in a reduction in tail tissue elongation.** (A) Representative images of ATS3:GAL4; UAS:GFP embryos and ATS3:GAL4;UAS:GFP;UAS:Kid embryos. Embryos were immunostained for GFP and activated Caspase 3. There is a clear reduction in GFP signal in embryos that have the UAS:Kid construct compared to those that do not. Insets show a clear increase in activated Caspase 3 in the spinal cord of UAS:Kid embryos. (B) Quantification of the relative number of Caspase 3-positive cells in a given region of the spinal cord in embryos with and without UAS:Kid shows that Kid toxin causes apoptosis in 16% of the spinal cord. (C) Representative examples of embryos at 30 hpf stained for nuclei and actin. ATS3:Kid embryos have a notable dorsal bend in the body axis compared to controls. The notochord is often kinked in the tail (asterisk). (D) Total somite counts shows no significant difference between ATS3:Kid and ATS3:GFP embryos. (E,F) Spinal cord length (E) and paraxial mesoderm length (F), measured from the 22nd somite, relative to total somite number, both show a significant decrease in ATS3:Kid embryos compared to ATS3:GFP controls. (G) The decrease in paraxial mesoderm length is consistent across all the tail somites. (H) Spinal cord length relative to mesoderm length from the 22nd somite shows that spinal cord elongation is more affected than paraxial mesoderm elongation in ATS3:Kid embryos. ATS3:Kid, *n*=20; ATS3:GFP, *n*=20. Conditions were compared using Mann–Whitney-Wilcoxon test. **P≤0.01; ***P≤0.001; ****P≤0.0001. ns, not significant. Relative length has units µm/somite.

Following the crossing between the ATS3:GAL4 and the UAS:Kid lines, we observed a clear effect on the morphogenesis of the body axis at the end of somitogenesis (Fig. 7A,C), which was comparable to the effect of ATS3 mutants (Wang et al., 2020). Ablation of ATS3^+^ cells caused a dorsal bending of the body axis that seemed similar to the dorsal bending of the tail observed in large neural progenitor ablations (Fig. 2C). As the ATS3:GAL4 driver also drives the expression of UAS:GFP in control (in crossed) and ablated (crossed to UAS:Kid) embryos, we could observe that much of the spinal cord was GFP^+^ in controls but there was a significant drop in GFP expression in UAS:Kid embryos (Fig. 7A). We observed increased levels of activated Caspase 3 (Fig. 7A, inset), confirming the successful action of the Kid toxin. Calculation of the average amount of Caspase 3 staining relative to neural tube nuclear density demonstrated that around 16% of neural tube cells were dying at this stage (Fig. 7B). Other tail tissues remained unaffected, although the notochord was consistently kinked in the tail, which could indicate that its full elongation is being prevented (Fig. 7C, asterisk).

We then compared tail formation between embryos from our control and ablated crosses at 30 hpf. We did not see an effect on the number of somites in the tail following genetic ablation of spinal cord cells (Fig. 7D). However, we did observe a decrease in spinal cord length, measured from the 22nd somite in the tail, in ablated embryos compared to controls (Fig. 7E). This demonstrates that the ablation of spinal cord cells affects the correct elongation of the tail spinal cord. We also found that there is a reduction in the length of the paraxial mesoderm in the tail (Fig. 7F), a decrease that is consistent across tail somites (Fig. 7G). This result demonstrates that elongation of the spinal cord secondarily affects the elongation of the paraxial mesoderm. By comparing the decrease in elongation of the spinal cord with the decrease in the elongation of the paraxial mesoderm, we showed that, similar to large two-photon ablations, the spinal cord is more affected than the paraxial mesoderm (Fig. 7H).

Despite the Kid-mediated ablation removing cells along the trunk, the effects of the lost cells on body axis elongation appeared to occur during tail formation. UAS:Kid embryos fixed at 20 hpf, when an average of 22 somites have formed, did not have a clear phenotype or a shorter body axis compared to controls and could only potentially be identified by a reduction in the UAS:GFP signal (Fig. S6A-C). Consistent with this, the effect of Kid expression on notochord development is only seen by 22 hpf (Labbaf et al., 2022).

To determine how the effect of Kid ablations relates to two-photon ablations, we first performed two-photon ablations in the trunk spinal cord anterior to the PSM at the 14-somite stage (Fig. S7A,C). Our initial ablations (size 1) were comparable to dorsal progenitor size 1 and 2 ablations (Fig. S7B). We also carried out larger ablations by performing a second size 1 ablation anterior to the first (Fig. S7B,C). At the end of somitogenesis, we observed a slight dorsal bending in the region of ablation, which was more pronounced following larger ablations (Fig. S7D). As with other ablations, loss of trunk spinal cord did not affect total somite number (Fig. S7E). Notably, ablation of trunk spinal cord did not affect the length of the tissues in the tail (Fig. S7F,G), indicating that any effects are local to the ablation region. We then measured the eight somites adjacent to each ablation (size 1, somites 14-21; size 2, somites 11-18) and found that larger trunk spinal cord ablations do cause a decrease in the length of the posterior trunk local to the ablation (Fig. S7H). This shows that the elongation of the trunk spinal cord is important for paraxial mesoderm elongation, although less so than the dorsal progenitors in the tailbud.

**Fig. 8.**
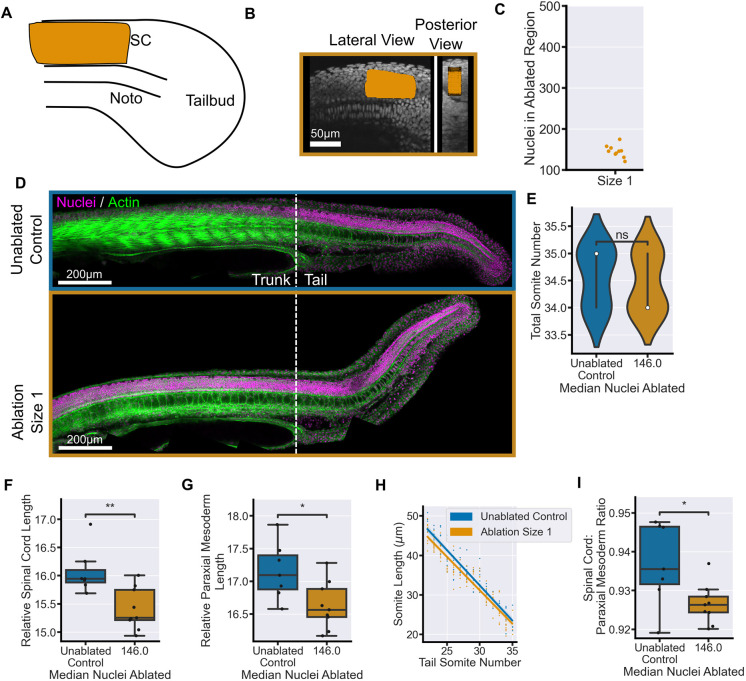
**Ablation of tail spinal cord cells leads to a reduction in tail tissue elongation.** (A,B) Schematic (A) and representative images (B) of the location of the spinal cord ablations (orange area) at the 14-somite stage. The ablations were performed in the spinal cord at the embryonic midline anterior to the notochord progenitors. (C) Number of nuclei in the ablation ROI prior to ablation showing a comparable number of nuclei ablated to other ‘size 1’ ablations. (D) Representative examples of embryos at 30 hpf stained for nuclei and actin. There is dorsal bending of the anterior part of the tail following spinal cord ablation. (E) Total somite counts are comparable between control and ablated embryos. (F,G) Spinal cord length (F) and paraxial mesoderm length (G) measured from the 22nd somite and relative to total somite number, both show a significant decrease in length in ablated embryos compared to controls. (H) Average somite length is prominently decreased in the anterior somites of ablated embryos compared to controls. (I) Spinal cord length relative to mesoderm length from the 22nd somite shows that there is a significantly greater reduction in spinal cord length compared to paraxial mesoderm length. Control, *n*=9; ablated, *n*=9. Conditions were compared using Mann–Whitney-Wilcoxon test. *P≤0.05; **P≤0.01; ns, not significant. Noto, notochord; SC, spinal cord. Relative length has units of µm/somite.

Finally, we used two-photon ablation to reduce cell number in the tail spinal cord (Fig. 8A). The ablation was localised in the midline of the spinal cord anterior to the start of the notochord progenitors, and therefore the tailbud (Fig. 8A,B). These ablations were comparable in size to the dorsal progenitor size 1 ablations (Fig. 8C; Fig. 2B). At the end of somitogenesis, ablated embryos had a dorsal bend to the tail (Fig. 8D). There was no difference in total somite number between control and ablated embryos (Fig. 8E). However, we did observe a significant decrease in relative spinal cord and paraxial mesoderm lengths in spinal cord-ablated embryos compared to controls (Fig. 8F,G). The effect on the paraxial mesoderm was more pronounced in the anterior somites of the tail (Fig. 8H), near where the ablation debris was located (Fig. 8D). However, as with the genetic ablation, the length of the spinal cord was more affected than the length of the paraxial mesoderm (Fig. 8I) so proportionality of the tissues was not maintained. This could be due to the bending of the tail affecting the balance of the two tissues.

Taken together, these data suggest that reduction in spinal cord cell number along its length effects the elongation of both itself and the adjacent paraxial mesoderm. The spinal cord is therefore a key driver of body axis elongation in zebrafish. However, the extent to which elongation is perturbed, and whether the balance of the tail tissues is maintained, depends on which region of the spinal cord or its progenitors is ablated.

## DISCUSSION

In summary, our results show that there is a capacity for the regulation of tail elongation, which ensures the proportional extension of the tail tissues. This demonstrates that the ability of the embryo to coordinate the scaling of tissue formation continues after pre-gastrulation stages whereby reduction in blastoderm size results in the complete scaling of all embryonic tissues (Almuedo-Castillo et al., 2018; Ishimatsu et al., 2018).

Notably, proportional regulation at pre-gastrulation stages occurs through changes in the pattern of cell differentiation through scaling of the signalling network (Almuedo-Castillo et al., 2018). In contrast, we demonstrate that the pattern of differentiation of the unspecified cells in the tailbud, the NMC cells, is not affected by the loss of neural-fated progenitors. Neither is there a compensatory shift in progenitor morphogenesis nor a compensatory increase in cell number. Consequently, ablation results in only a reduction of the number of the dorsally located NMC cells in the tailbud. It is notable that the progression of NMC cell differentiation is not dependent on the overall number of progenitors, which fits with previous work demonstrating that NMC cells generate their own permissive signalling environment (Martin and Kimelman, 2008; Bouldin et al., 2015).

The lack of any widespread NMC cell response to ablation is in contrast to the loss of tissue proportions observed when manipulating Wnt signalling at tailbud stages (Martin and Kimelman, 2012). This raises an important distinction between what a cell can do and what a cell will do in the context of its environment. A similar distinction has been previously noted in relation to NMC cell behaviour and global division levels (Wymeersch et al., 2021; Sambasivan and Steventon, 2021). In this case, the strong dorsal-ventral flow of cells in the tailbud (Lange et al., 2024; Lawton et al., 2013) is not sufficiently disturbed by the ablation and therefore does not result in a large-scale change in the contribution of NMC cells to neural versus mesodermal fate in the short or long term following ablation. A similar process of diverging cell flows has been proposed to split neural- and mesodermal-fated NMC cells in the early chick embryo (Wood et al., 2019 preprint).

Despite progenitor dynamics being highly robust to ablation, the elongation of the tail is sensitive specifically to the number of neural progenitors in the tailbud or cells in the spinal cord. In contrast, paraxial mesoderm elongation is not so easily affected by the loss of a similar number of mesoderm progenitors. This suggests that the major contribution to proportional tissue elongation does not come from a significant re-distribution of NMC cells within the tailbud. Instead, our results point towards the importance of spinal cord extension for correct tail elongation. The spinal cord undergoes the most volumetric growth of all tail tissues, whereas the paraxial mesoderm elongates through thinning and lengthening (Steventon et al., 2016). Consequently, loss of dorsal progenitors as the raw material for volumetric growth has an outsized effect on spinal cord formation. Reduced spinal cord elongation, in turn, affects the elongation of the paraxial mesoderm and thus maintains tissue proportions. Even when exact proportionality between the tissues is lost, a reduction in spinal cord or dorsal progenitor number consistently affects paraxial mesoderm elongation. Whether this effect is transmitted through the mechanical coupling of the extracellular matrix, as has recently been described in several works (Tlili et al., 2019; Guillon et al., 2020; Dray et al., 2013), or transmitted through chemical signals between the two tissues, will be an important avenue for future study. Together, this positions spinal cord formation as a driver of posterior body elongation in zebrafish.

Finally, our results demonstrate the importance of considering the effect of multi-tissue tectonics (Busby and Steventon, 2021) on developmental processes. It is important to note that the exact interactions between the tissues is likely to vary across developmental time, space, and evolution so that the contribution of the morphogenesis of each tissue to posterior body elongation is not static or absolute. Notochord elongation may play a more prominent role at later stages (McLaren and Steventon, 2021), whereas in avian species for example, the morphogenesis and growth of the PSM has an effect on the elongation of the spinal cord (Romanos et al., 2024; Xiong et al., 2020). Overall, this work contributes to an expanding body of evidence that the formation and growth of the tail tissues, rather than the action of the progenitors themselves, are a major driver of posterior body elongation in zebrafish (McLaren and Steventon, 2021; Tlili et al., 2019; Özelçi et al., 2022; Guillon et al., 2020).

## MATERIALS AND METHODS

### Animal husbandry

The maintenance of adult zebrafish, including any regulated procedures, was conducted in accordance with the Animals (Scientific Procedures) Act 1986 Amendment Regulations 2012, following ethical review by the University of Cambridge Animal Welfare and Ethical Review Body (AWERB). Standard E3 media was used to culture embryos in all experiments except for those involving lightsheet microscopy in which Methylene Blue was omitted. Embryos were staged according to Kimmel et al. (1995). To prevent involuntary embryonic muscle contraction, tricaine (ethyl 3-aminobenzoate; Sigma-Aldrich, A-5040) was added at 0.16 g/ml in E3 medium. Transgenic zebrafish lines used were: *Tg(actb2:H2a-mCherry)*, *Tg(actb2:H2b-GFP)*, *Tg(actb2:Lifeact-EGFP)* (Behrndt et al., 2012), *Tg(sox2:2a-sfGFP)* (Shin et al., 2014), *Tg(β-actin:myl12.1-mCherry)* (Maître et al., 2012), and *Tg(7xTCF-Xla.Sia:GFP)* (Moro et al., 2012). Wild-type zebrafish strains used were: AB, TL (University of Cambridge, UK).

### Microscopy

#### Two-photon microscopy

Embryos were mounted as described by Hirsinger and Steventon (2017). Embryos were imaged and ablated, using a TriM Scope II upright two-photon microscope (LaVision Biotec) with a 25× (1.05 NA) water immersion lens. For ablation, the laser was set to 900 nm and the imaging window was set to 200×200 in order to achieve a dwell time greater than 9 µs. Laser power ranged from 1.3 to 0.5 W during the course of experiments, and ablation was carried out with 80% laser power. The embryo was imaged for at least one complete stack prior to and after ablation. A successful ablation was determined by the presence of a small amount of recoil of adjacent nuclei at the time points following ablation. Ablation location was determined based on the morphology of the tailbud. Neural progenitor ablations were localised to the dorsal-posterior wall, which is located posterior and dorsally to the notochord progenitors and floor plate. This arc corresponds to the arc of *sox2^+^/tbxta^+^* cells in this region. The ablations were centred on the midline in the medio-lateral axis as denoted by the notochord. Size 1 ablations were 20 μm deep in this axis. Size 2 ablations were 30 μm deep and size 3 ablations were 40 μm deep. Size 4 ablations were also 40 μm deep but extended further anterior into the neural progenitors. Mesoderm progenitor ablations were localised to the lateral aspect of the tailbud closest to the objective, ensuring that they were lateral to the notochord progenitors. They were localised to the posterior of the tailbud and ventral to the boundary between the PSM and the spinal cord. This included the location of the lateral extension of the *sox2^+^/tbxta^+^* cells. Size 1 ablations were 20 μm deep in the medio-lateral axis, size 2 and 3 ablations were 40 μm deep. Size 3 ablations extended more anteriorly into the PSM. Trunk spinal cord ablations were performed at the 14-somite stage anterior to the PSM boundary in the midline of the spinal cord 20 μm deep. Larger spinal cord ablations were performed with the addition of another ablation anterior to the first, both at 20 μm deep. Tail spinal cord ablations were performed at the 14-somite stage anterior to the beginning of the notochord progenitors at the spinal cord midline, 20 μm deep.

#### Lattice lightsheet microscopy

A V-shaped mould was created using 1% agarose in a 35 mm glass-bottomed dish. Individual embryos were aspirated in 1% low melting point agarose and placed in the bottom of the V-shaped mould. An eye-lash tool was used to orient the embryo ventrally so that the tailbud was pressed against the bottom of the dish. Once the agarose set, the embryo was covered with E3 medium and tricaine. The embryos were then imaged on a Lattice Lightsheet 7 microscope (Zeiss) using inverted water immersion single illumination (13.3×; NA 0.4) and detection (44.83×; NA 1.0) objectives. A full stack was taken every 30 s at 28°C. Following imaging, embryos were removed from agarose and allowed to grow at 28°C in E3 media overnight to confirm that mounting did not have an adverse effect on embryo growth or morphology.

#### Confocal microscopy

Fixed embryos were dissected completely away from the yolk and the head removed to ensure they lay flat on their lateral axis. They were mounted in VectaShield (Vector Laboratories) mounting media between two 1H coverslips stuck together with double-sided tape. All fixed embryos were imaged on an inverted Zeiss LSM700 confocal microscope. For embryos at 30 hpf, the whole embryo was imaged in sections using a 20× objective, with 4× line averaging. Images of the tailbud were taken using a 40× objective, with 2× line averaging. Images were collected with the same laser intensities, gain, and pixel resolution for each experiment.

### Fixing and staining

Embryos were fixed in 4% paraformaldehyde for 1 h on the bench or up to 24 h at 4°C. Embryos were then washed into PBS without magnesium and calcium ions with 0.05% Tween [PBST (−/−)]. Nuclei were stained with 1:500 DAPI and actin was stained with 1:1000 phalloidin tagged with Alexa Fluor 647 nm in PBST (PBS with 0.05% Tween) for at least 24 h. Individual two-photon-ablated embryos were stained separately to be able to link the ablation with the final morphology. To visualise mRNA or proteins, *in situ* embryos were pooled into ablated and control groups following ablation. Fluorescence *in situ* hybridisation was performed using the hybridisation chain reaction method as described by Choi et al. (2018). Immunohistochemistry was carried out as described by Sorrells et al. (2013). Primary antibodies used were: chicken anti-GFP (1:200; Abcam; Ab13970; RRID: AB_300798), rabbit anti-activated Caspase 3 (1:500; Abcam; Ab13847; RRID: AB_443014), and mouse anti-β-Catenin (1:200; Sigma-Aldrich; C7207; RRID: AB_476865). All secondary antibodies were raised in goat. Secondary antibodies were used at a concentration of 1:1000. Secondary antibodies used were: anti-mouse Alexa Fluor A488 (Thermo Fisher Scientific, A32723; RRID:AB_2633275), anti-rabbit Alexa Fluor A633 (Invitrogen, A21071; RRID:AB_2535732), anti-chicken Alexa Fluor A488 (Invitrogen, A-11039; RRID: AB_2534096). DAPI was added at the end of the protocol at 1:500.

### Genetic ablation

ATS3:GAL4 (Wang et al., 2020), which also contained a UAS:GFP cassette, were crossed with UAS:Kid; (Labbaf et al., 2022) or simply in-crossed to produce embryos ATS3:GAL4;UAS:GFP;UAS:Kid (ablated’) and ATS3:GAL4;UAS:GFP (‘control’) embryos. This resulted in expression of GFP or GFP and bacterial Kid toxin in AdamTS3-expressing cells of the spinal cord. Embryos were selected based on phenotype.

### Photolabelling

Photolabelling was performed using the photoconvertible Kikume protein (Hatta et al., 2006). The plasmid nls-Kikume (from Ben Martin, SUNY Stony Brook, NY, USA) was used to generate mRNA. RNA was synthesised using the mMessage-mMachine kit from Thermo Fisher Scientific (AM1344). Embryos were injected prior to the first cell division with a 100 µm diameter droplet of 300 ng/µl Kikume mRNA. For photoconversion, embryos were mounted as described by Hirsinger and Steventon (2017). Photoconversion was performed with a 30 s exposure of a 405 nm laser on a Zeiss LSM700 confocal microscope using a circular ROI with an average diameter of 70 µm. The ROI was placed in the ventral and posterior most part of the tailbud just prior to the 14-somite stage. The initially green light emission from the fluorophore (KikG) becomes red light emission (KikR) as the protein photoconverts. Following photoconversion, ablations were performed as described and the embryos were imaged at 2.5 h post-ablation and 8.5 h post-ablation (end of somitogenesis) using the Zeiss LSM700 confocal microscope.

### Image analysis

#### Visualisation and registration

Images were visualised using Fiji (Schindelin et al., 2012), or Napari (https://napari.org/stable/; available at https://zenodo.org/records/7276432). Two-dimensional length measurements were made using Fiji's segmented line ROI tool. For measurements of tail length, images were collected individually and stitched together using a bespoke script for successive rounds of pairwise stitching utilising the Fiji Stitching plugin (Preibisch et al., 2009). Where necessary, images were rotated using the TransformJ plugin (Meijering et al., 2001). The segmented line tool was used to measure the length of each tissue. The spinal cord was measured from the 22nd somite until the tip of the spinal cord along the floor plate. The tip of the spinal cord was clearly visible at these stages based on where the phalloidin-stained lumen ends. The paraxial mesoderm was measured from the 22nd somite down the middle of each somite and then down the middle of the PSM to the centre of the tip of the tailbud (not including ectoderm). At these stages, the remnants of the tailbud only contribute to mesodermal structures. The individual somite lengths and PSM lengths were calculated using the distance between the points on the segmented line. Lengths were normalised by dividing by total somite number to adjust for small differences in the developmental timing. Registration of tailbud point clouds was performed using ZebReg as described by Toh et al. (2022). A radius of 11 µm was used to average adjacent nuclei across embryos as within a single embryo; this gives an average of 14 nearest neighbours, i.e. the number of neighbours in a perfect lattice. Data were visualised using the Python 3.9 packages Matplotlib and Seaborn. Statistical analysis was performed using the packages Scipy.Stats and StatAnnotations. Box plots are set to display the median and quartiles of the data. Line plots show the mean of the data and the 95% confidence interval. The functions used in image analysis can be viewed on GitHub https://github.com/DillanSaunders/TailbudAnalysis.

#### 3D nuclear segmentation

In order to quantify nuclei number and gene expression, a bespoke 3D segmentation pipeline was developed. Prior to segmentation of nuclei, images were pre-processed. First, intensities were equalised using adaptive histogram equalisation with a 3D kernel cube of length 10 µm (based on average nuclear diameter). Second, the images were filtered using a difference of Gaussian filter (low sigma=1; high sigma=3) and thresholded at 0. Segmentation was performed in two steps. First, the nuclei were segmented in 2D using the StarDist 2D default model (Schmidt et al., 2018 preprint). 2D labels were then joined in 3D using a threshold of 0.6 intersection over union using a function adapted from CellPose (Stringer et al., 2021). The HCR images of fluorescent mRNA were then pre-processed using two rounds of median filtering (kernel=0.8 µm^3^, based on average size of nuclear transcription spot). The median intensity values for *sox2* in the notochord and *tbxta/tbx16* in spinal cord were used as an estimation of background intensity for each channel. This was used to filter out nuclei with background levels of both genes. The nuclei of the hypochord that were also *sox2*^+^*/tbxt*^+^ were then manually removed by eye. For quantification in the spinal cord and PSM at the end of somitogenesis, morphological criteria were used. The anterior PSM region was manually isolated between the nascent somite and the beginning of the notochord progenitors. The posterior of the spinal cord was manually isolated between the anterior PSM boundary and the posterior tip of the spinal cord.

#### Cell tracking

Images acquired from the Zeiss lattice lightsheet microscope were saved into a single .czi file. These files were opened in ZenBlue software, cropped to the correct size and re-saved as .czi files. As the tail is prevented from growing outwards during this time, these images do not need global registration. The images were opened in Fiji and converted to HDF5 data format (with deflate compression) using the plugin BigDataViewer (Pietzsch et al., 2015). Following conversion to HDF5/XML format, the lightsheet movies were opened in the Fiji plugins MaMut (Wolff et al., 2018) or Mastodon (https://github.com/mastodon-sc/mastodon). MaMut was used to generate a sample of manual tracks to be able to parameterise automatic tracking accurately in Mastodon. Automatic spot detection used a diameter of 10 µm with a quality threshold of 50. Automatic spot linking was performed using a displacement of 10 µm and a frame gap of no more than two frames. Cell division was permitted with the distance set to 15 µm. Metrics of cell motion are well established in the field of cell migration and were calculated as described previously (Lawton et al., 2013; Beltman et al., 2009; Hu et al., 2023).

## Supplementary Material



10.1242/develop.204438_sup1 Supplementary information
