# Supplementary material for: Spinal cord elongation enables proportional regulation of the zebrafish posterior body
Source: Development. 2025 Jan 9;152(1):dev204438. doi: 10.1242/dev.204438 (PMC11829759; doi:10.1242/dev.204438)
Supplement: Supplementary information [file develop-152-204438-s1.pdf]

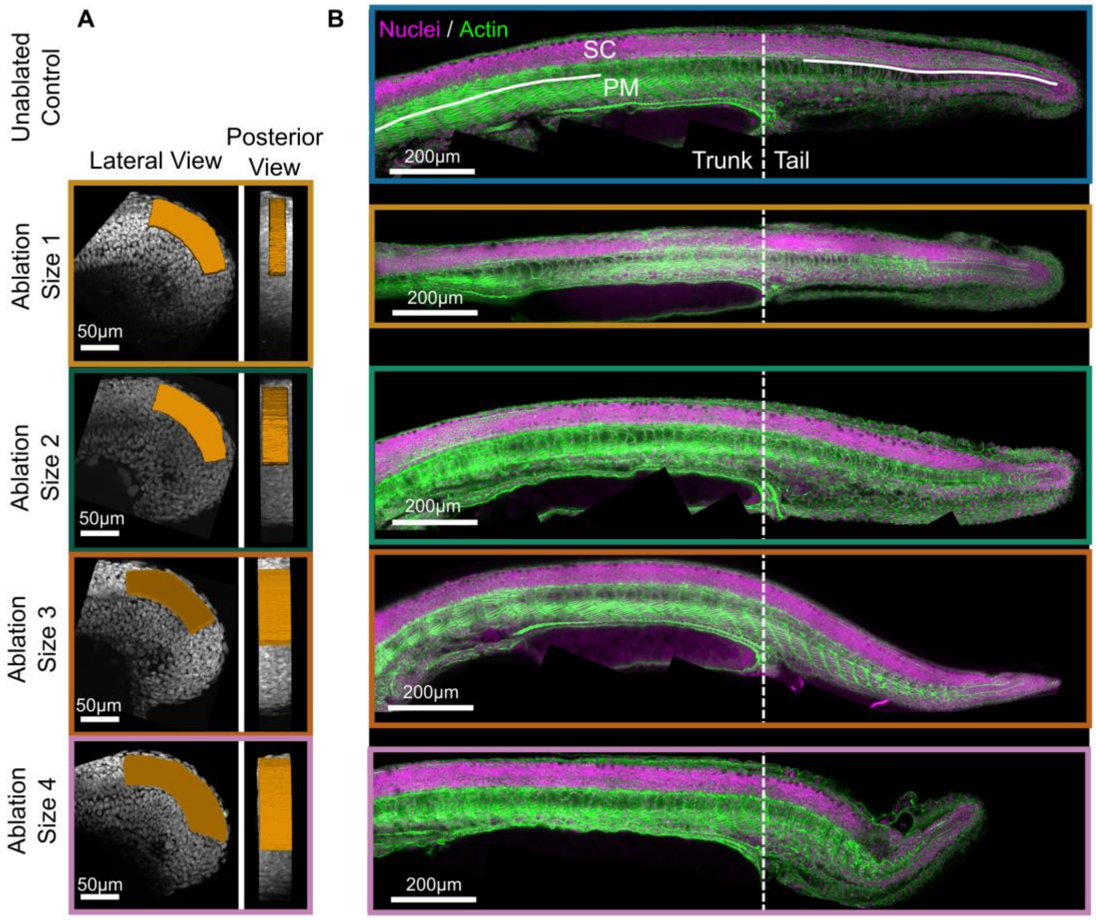

Fig. S1. Dorsal progenitor ablation sizes and locations. (A) Representative images of the four classes of dorsal progenitor ablation size. Images are maximum intensity 3D renderings from Napari. The ablated region is visualised as an iso-surface (orange). All ablation sizes occupy a similar location in the dorsal progenitors. Size was increased by increasing the depth of ablation, always centred on the embryonic midline. Size 3 and 4 ablations have the same medio-lateral depth, but size 4 ablations extend further towards the neural tube. (B) The resulting body axis phenotypes at 30hpf fixed and stained with DAPI and Phalloidin. Size 1 and 2 ablations are comparable in morphology to control embryos while size 3 and 4 ablations display dorsal tail bending and spinal cord abnormalities.

Development • Supplementary information

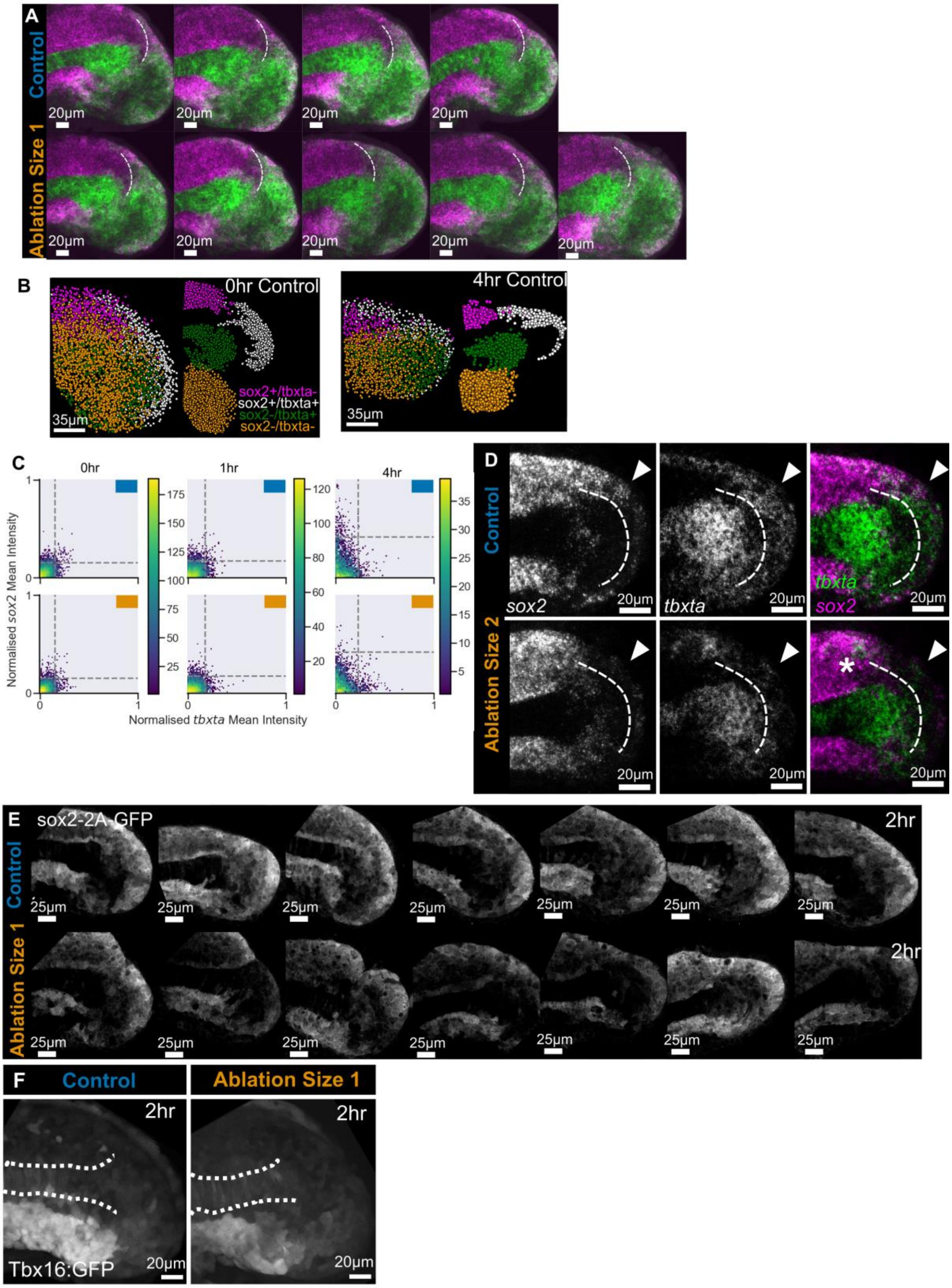

Fig. S2. Dorsal progenitor ablation does not affect the gene expression pattern in the tailbud. (A) Mean (average) midline projections of control and ablated embryos at 1hr post ablation stained for *sox2* and *tbxta* mRNA. The *sox2/tbxta* gene expression pattern shows variability between control embryos as well as ablated

Development • Supplementary information

embryos, an example being the anterior boundary of *sox2/tbxta* co-expression in the dorsal progenitors (dashed line). (B) Centroids for each nucleus of 3D segmented 0hr and 4hr control embryos. Using the mean expression of *sox2* and *tbxta* different cell populations within the tailbud can be isolated. NMC nuclei are shown in white. (C) Heatmaps of normalized expression levels of mean nuclear *sox2* and *tbxta* across all embryos at each timepoint. In control embryos the NMC cells start off with balanced levels of *sox2* and *tbxta,* by 4hrs mean expression levels have spread and there are more cells with comparatively higher levels of either gene. Gray dashed lines indicate the 95th percentile of the control data to highlight the spread over time. The vast majority of NMC cells in ablated embryos follow the same pattern as the control embryos but there are fewer cells which reach such high levels of *sox2* and fractionally more that have higher levels of *tbxta*. The heatmap is generated using bins of 0.0125 width. (D) Mean (average) projections of control and ablated embryos at 4hrs after size 2 ablations. Here the tailbud is noticeably smaller and the NMC region (arrows) is depleted but there are still some *sox2/tbxta* expression present in the posterior wall. Disorganised expression associated with cell death is present (asterisk). Control, n=4; ablated, n=5. (E) Sox2-GFP transgene expression visualised with GFP antibody shows expression in the posterior wall and spinal cord 2hrs after ablation, similar to controls. The exact pattern is variable between control embryos, as well as ablated embryos. Ablated, n=7; control, n=7 (F) Tbx16-GFP transgene expression visualised with GFP antibody shows a comparable pattern between control and ablated embryos. Ablated, n=5, control, n=5.

Development • Supplementary information

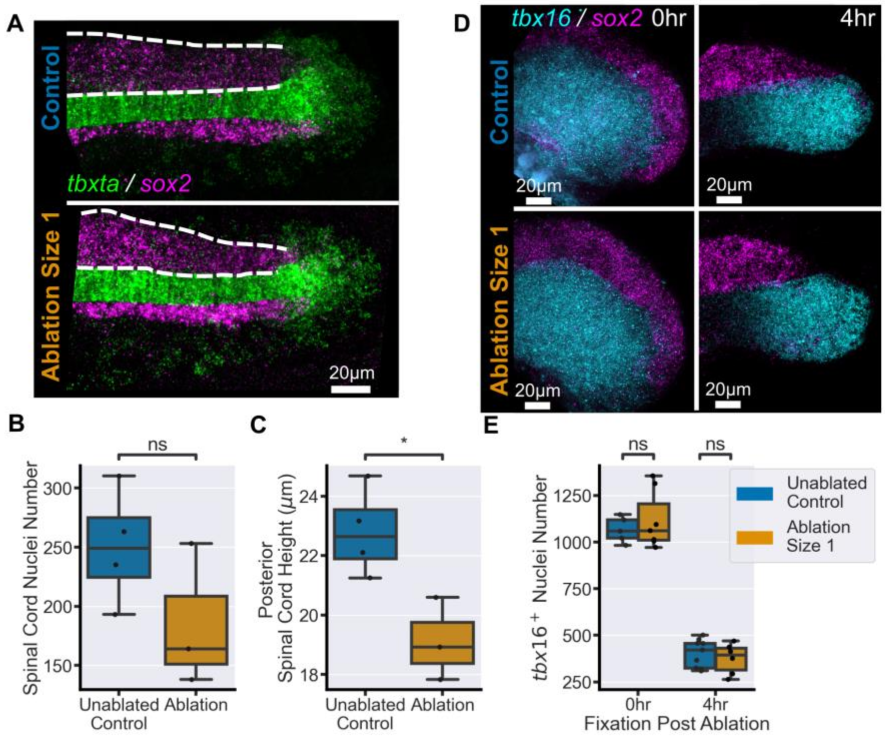

Fig. S3. Dorsal progenitor ablation does not affect mesodermal progenitor number. (A) Representative maximum projections of the tip of the tail at the end of somitogenesis (8hrs post ablation) in control and dorsal progenitor ablated embryos. The spinal cord is qualitatively reduced in height (dashed lines) in ablated embryos. (B) Quantification of nuclei number in the spinal cord from the anterior boundary of the PSM to the posterior tip of the spinal cord. There is no significant difference according to the statistical test but 2 out of 3 embryos have a reduced number of nuclei. (C) All three ablated embryos have a posterior spinal cord that is reduced in height compared to controls as measured immediately anterior to the notochord progenitors. Control, n=3; ablated, n=3. (D) Representative examples of control and ablated embryos 0hrs and 4hrs after dorsal progenitor ablation, stained for *tbx16* and *sox2* mRNA. The *tbx16* domain (mesodermal progenitors) is qualitatively comparable between control and ablated embryos. (E) Quantification of *tbx16* nuclei number at 0hr and 4hrs post dorsal progenitor ablation. There is no significant difference in the number of nuclei in control and ablated embryos at either timepoint. 0hr: control, n=5; ablated, n=7. 4hr: control, n=5, ablated n=5. Conditions were compared using Mann-Whitney-Wilcoxon test. *, p <=0.05.

Development • Supplementary information

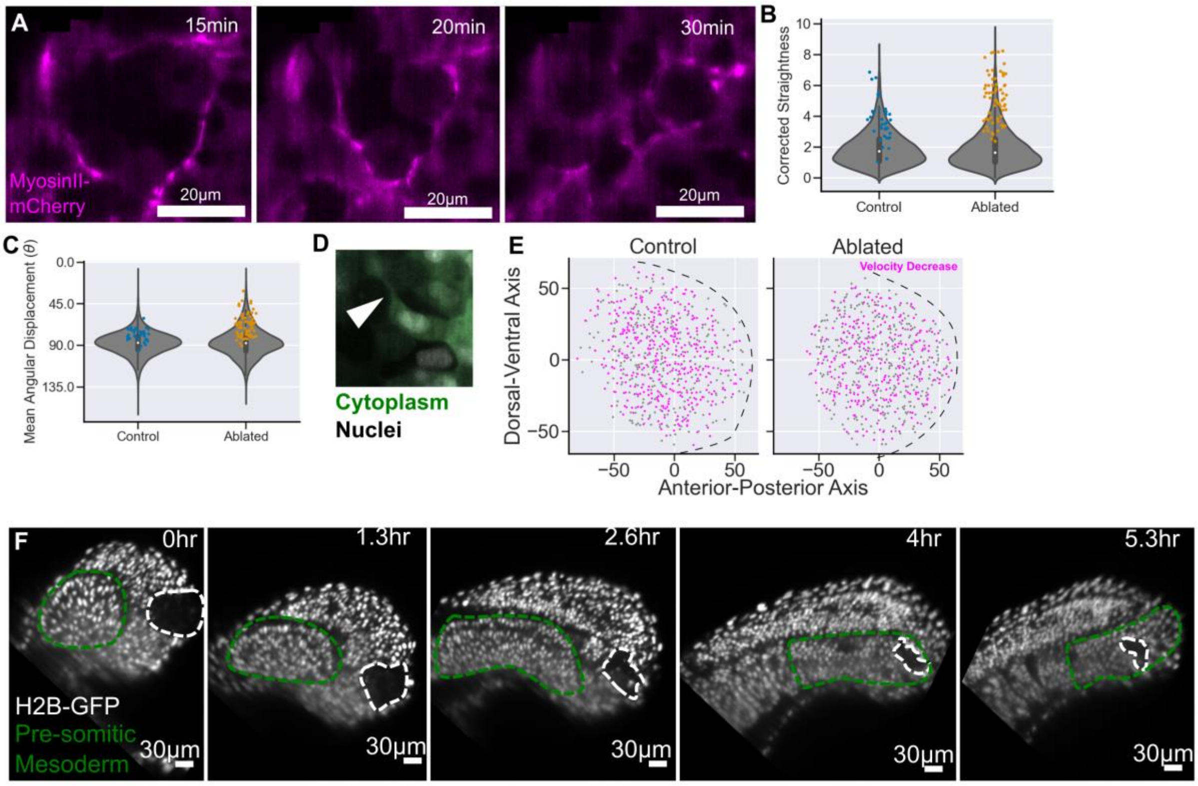

Fig. S4. Dorsal progenitor ablation does not perturb global cell movements. (A) Representative images of ablation healing visualised using MyosinII:mCherry. Cells increase myosin levels at the ablation edge until the ablation closes up. Cell tracks which have a high mean squared displacement exponent (see Fig 4E) are shown as coloured points overlaid on the violin plots of all other tracks within 60μm of the ablation centre. These tracks also rank highly, compared to all tracks (grey), in other measures of directional motion, (B) corrected track straightness, and (C) mean angular displacement. (D) Some directional tracks are associated with cells moving into the ablated region (arrow indicates cell protrusion crossing the ablated region). (E) The dorsal-ventral and anterior-posterior localisation of tracks exhibiting the greatest amount of velocity decrease (lower quartile). There is no bias in velocity decrease associated with the dorsally located progenitors in either control or ablated embryos over this timescale. (F) Long-term live imaging of the tailbud following the healing of a large ablation. Over the course of 5hrs what remains of the ablated debris moves from the dorsal-posterior of the tailbud into the PSM rather than the spinal cord.

Development • Supplementary information

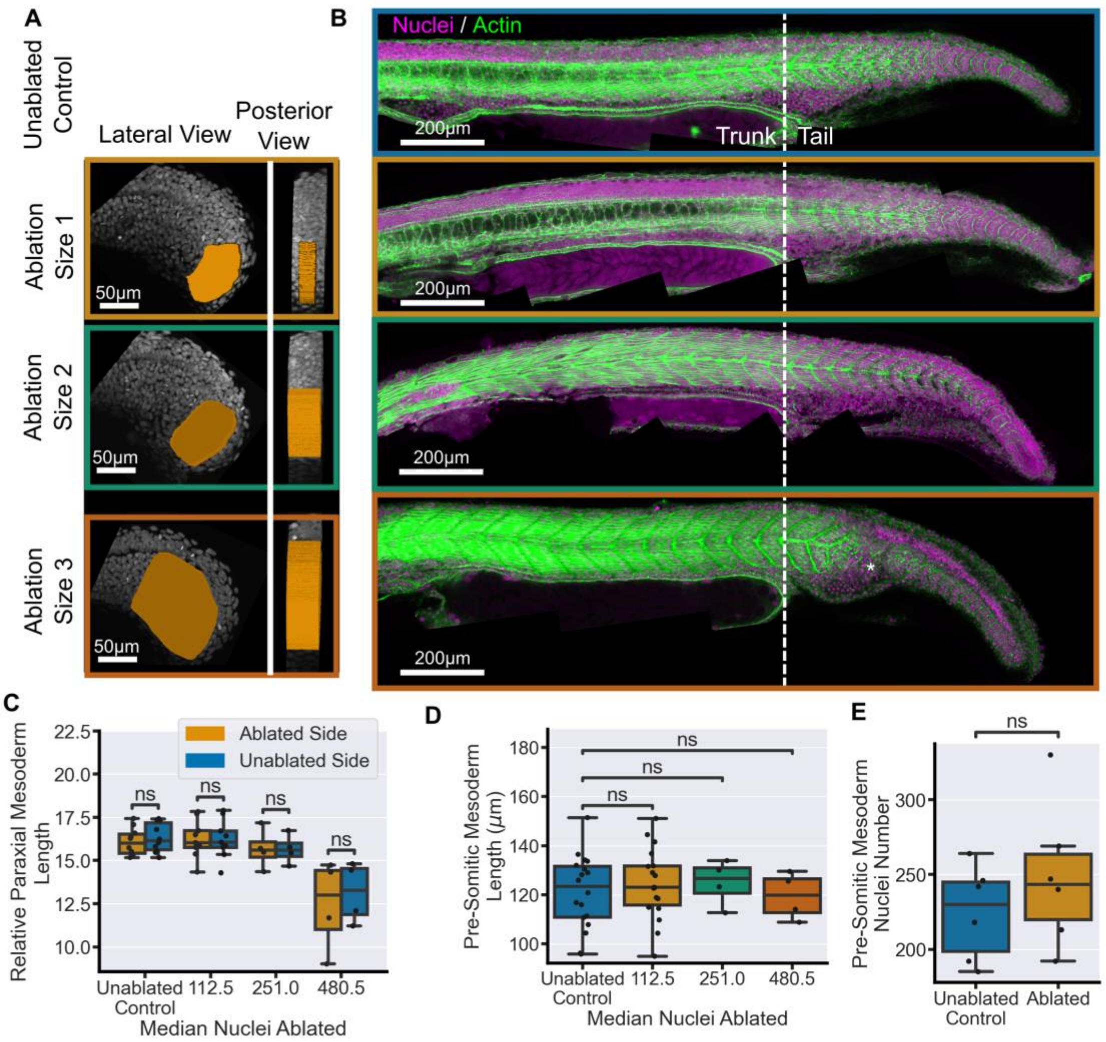

Fig. S5. Mesoderm progenitor ablation does not affect tissue elongation. (A) Representative images of the three classes of mesoderm progenitor ablation size. Images are maximum intensity 3D renderings from Napari. The ablated region is visualised as an iso-surface (orange). All ablation sizes target a similar location, the posterior paraxial mesoderm progenitors located laterally to the notochord progenitors. Size was increased by increasing the depth of ablation, ensuring that it did not get too close to the midline. Size 2 and 3 ablations have the same medio-lateral depth, but size 4 ablations extend further towards anterior of the PSM. (B) The resulting body axis phenotypes at 30hpf fixed and stained with DAPI and Phalloidin. Size 1 and 2 ablations are comparable in morphology to control embryos while size 3 ablations display paraxial mesoderm abnormalities. (C) Ablations were only performed on one side of the paraxial mesoderm, the contralateral side was also measured and the paraxial mesoderm length of the two sides compared. There

Development • Supplementary information

is no significant difference between the length of the ablated and unablated sides at any size of mesoderm progenitor ablation however where ablations do strongly affect paraxial mesoderm length they may do so more on the ablated side. (D) PSM length is consistent between controls and ablated embryos of all sizes. (E) Quantification of nuclei number left in the anterior PSM at the end of somitogenesis (30hpf) shows that there is no difference between control and size 1 ablated embryos. Suggesting the effect of ablation is distributed across the paraxial mesoderm. Control, n=6, ablated, n=6. Conditions were compared using Mann-Whitney-Wilcoxon test. Relative length has units of μm/somite.

Development • Supplementary information

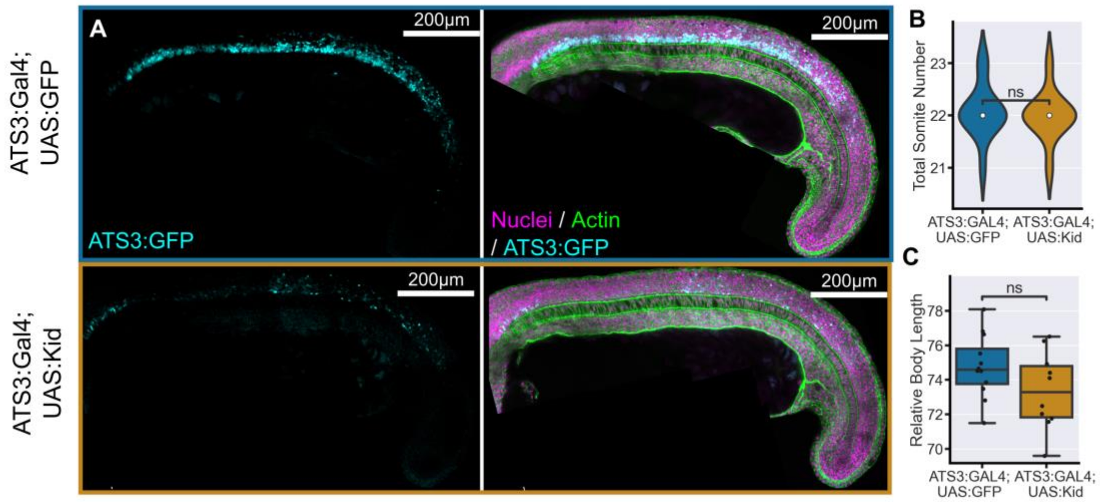

Fig. S6. Cell death throughout the spinal cord primarily effects body elongation during the formation of the tail. (A) Representative embryos from crosses between ATS3:GAL4;UAS:GFP fish and between ATS3:GAL4;UAS:GFP and UAS:Kid fish. Not all embryos from the UAS:Kid cross will have the desired genotype. At 20hpf (early tail elongation stage) embryos with the UAS:Kid transgene cannot be identified by phenotype as they are morphologically indistinguishable from controls. Instead embryos with weak GFP fluorescence in the spinal cord were selected from the UAS:Kid cross (based on the weaker GFP fluorescence we see when the phenotype is evident, see Fig 7) and compared to embryos from the UAS:GFP line without the Kid toxin. (B) Comparison of total somite number between the UAS:Kid and UAS:GFP control groups shows no significant difference and an average of 22 somites. (C) Body length, relative to somite number, from the first somite to the tip of the tail was measured for both suspected UAS:Kid embryos and UAS:GFP only embryos and though some weak fluorescent embryos do have a shorter relative body length compared to controls this difference is not significant. Conditions were compared using Mann-Whitney-Wilcoxon test. Relative length has units of μm/somite.

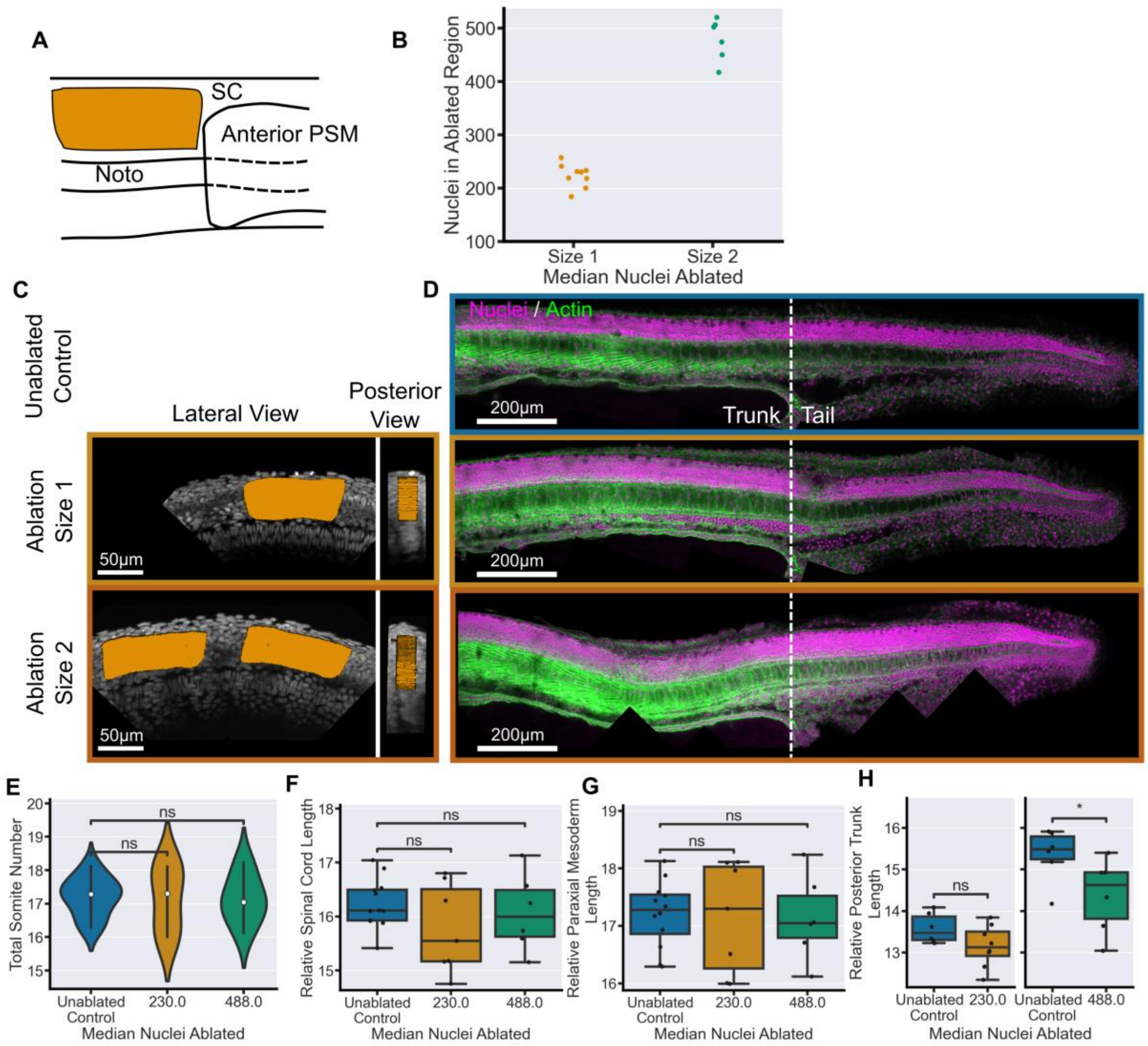

Fig. S7. Ablations of trunk spinal cord have a local impact on body elongation. (A) Schematic showing the localisation of the size 1 trunk spinal cord ablations which were performed in the spinal cord immediately adjacent to the anterior margin of the PSM at the 14 somite stage. (B) Number of nuclei in the ablation ROI prior to ablation with increased ROI size. (C) Representative images of the two classes of trunk spinal cord ablation sizes. Images are maximum intensity 3D renderings from Napari. The ablated region is visualised as an iso-surface (orange). All ablation sizes target a similar location, anterior to the PSM at the 14 somite stage. The size 2 ablations were made larger by immediately performing a second size 1 ablation anterior to the first. (D) The resulting body axis phenotypes at 30hpf fixed and stained with DAPI and Phalloidin. Both size 1 and size 2 ablations have a slight dorsal bend in the anterior tail or trunk. This is more pronounced in the size 2 ablated embryos. (E) Trunk spinal cord ablations do not affect total somite number. The length of the (F) spinal cord, and (G) paraxial mesoderm, relative to somite number

Development • Supplementary information

were measured from the 22nd somite to the tip of the tail and there is no significant difference between control and ablated embryos. This indicates that ablation of trunk spinal cord does not affect the elongation of the tail. (H) Measurement of the trunk somites adjacent to the spinal cord ablations (size 1, somites 14-21; size 2, somites 11-18), relative to somite number, show that size 1 ablations do not have a significant effect on paraxial mesoderm elongation, but size 2 ablations do.

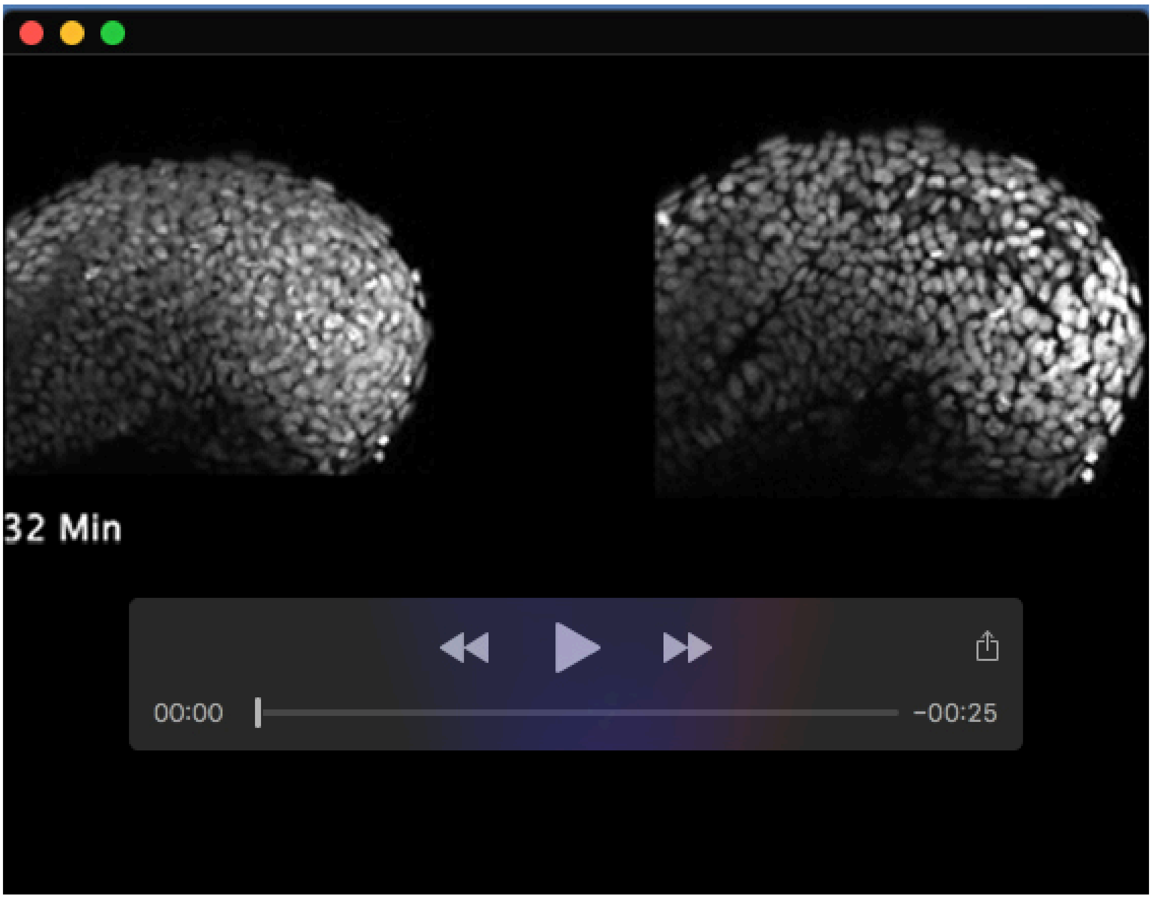

Movie 1. Production of nuclear debris following ablation healing. Two views of a tailbud imaged immediately after ablation on a two-photon microscope using H2B-GFP transgenic line to mark nuclei. The first panel is a maximum projection of several lateral slices of the tailbud to visualise the pre-somitic mesoderm. The second panel is a maximum projection of several medial slices of the tailbud to visualise the dorsal progenitors. The ablation location can be observed by the decreased fluorescence intensity in dorsal region of the tailbud in the second panel. Nuclear debris is first observed after 1 hour and becomes prevalent around 90 minutes in the dorsal medial region of the tailbud. The debris moves rapidly and is then observed at high amounts in the lateral PSM by 100 minutes.

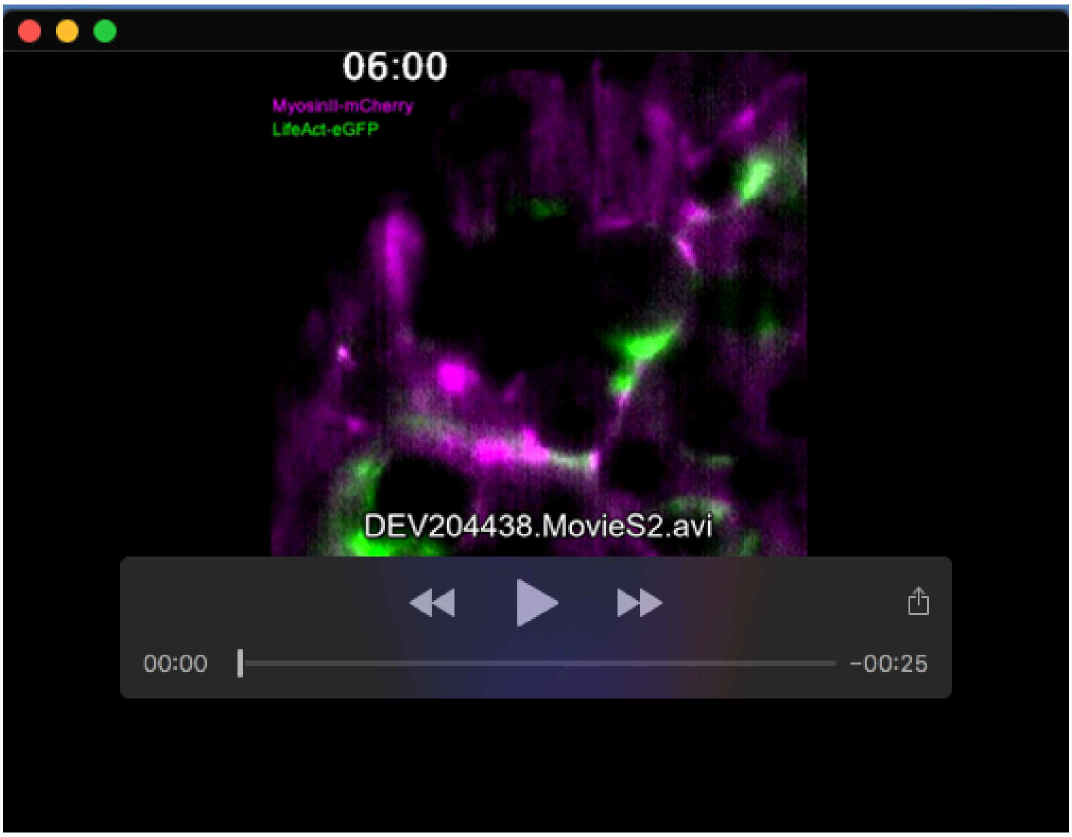

Movie 2. Increased acto-myosin activity during ablation healing. Live imaging of MyosinII-mCherry and LifeAct-eGFP transgenic lines following dorsal progenitor ablation. MyosinII and Actin activity is increased at the edge of the ablated region (centre). By the time ablation has healed acto-myosin activity is reduced in the ablated area. Time step 30s.

Development • Supplementary information

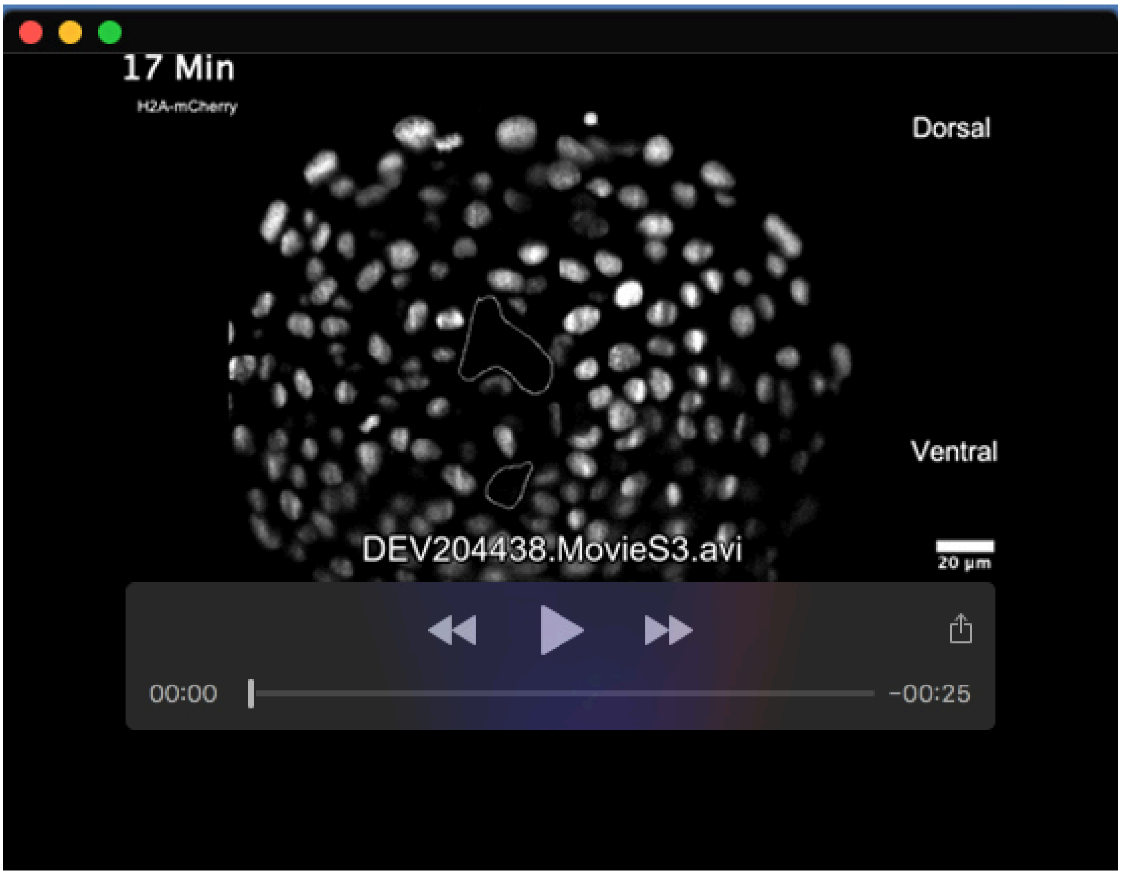

Movie 3. Global dorsal to ventral flow during ablation healing. Lattice lightsheet live imaging of H2A-mCherry transgenic line to visualise nuclei. The ablated region has been manually outline in white at each timepoint and can be seen to heal over the course of the movie. During this time the predominant flow of cells, and movement of the ablation, is from dorsal towards ventral. Movies such as this were used to generate tracking data. This is a posterior view of the tailbud. Time step 1 minute.
