# Supplementary material for: Spinal cord elongation enables proportional regulation of the zebrafish posterior body
Source: Development. 2025 Jan 9;152(1):dev204438. doi: 10.1242/dev.204438 (PMC11829759; doi:10.1242/dev.204438)
Supplement: Supplementary file 1 [file develop-152-204438_review_history.pdf]

Spinal cord elongation enables proportional regulation of the zebrafish posterior body

Dillan Saunders, Carlos Camacho-Macorra and Benjamin Steventon
DOI: 10.1242/dev.204438

Editor: James Briscoe

Review timeline
Submission to Review Commons: 1 April 2024
Submission to Journal of Cell Science: 8 October 2024
Accepted: 15 November 2024

Reviewer 1

Evidence, reproducibility and clarity

This study uses laser ablations to study the mechanism of posterior body elongation in zebrafish. The authors ablate cells in the tailbud and examine the behavior of a bipotential population of cells (NMPs or NMCs) which can give rise to both spinal cord and paraxial mesoderm. The authors do not detect a change in fates of the NMP cells but without long- term fate mapping, it is difficult to make firm conclusions about cell fates. That said, the authors see clear differences in phenotypes when ablating neural or mesodermal progenitors with the form producing larger effects on elongation. A final set of experiments that genetically ablates spinal cord cells also lead to a shortened body axis. Generally speaking, the hypothesis that spinal cord elongation drives posterior body elongation is supported by the data. However, in many cases, it is difficult to draw unambiguous conclusions from the data as they are presented.

Major comments:

In Figure 3A, on the 1hr ablation panel, it looks like the ablated region has more tbxta expression. Is this a consistent result?

Perhaps analyzing tbxta and sox2 expression using intronic probes to visualize nascent transcription would be a more sensitive assay for changes in NMP activity on the short time scales of these experiments.

In Figure 3B, it looks like elevated Sox2 expression in the ventral tailbud after ablation. Wouldn't this suggest that more NMPs differentiate as mesoderm?

The data in Figure 5C-H are not compelling. The conclusion is that there is no change in Wnt signaling. Maybe. There are no apparent changes, but what are the resolution of the data? How sensitive is the TCF::GFP transgene to changes in endogenous levels of Wnt signaling?

In figures 6E-G, are the paraxial mesoderm measurements made on the ablated side or unablated side?

It is difficult to interpret the data in Figure 6 as only one side of the paraxial mesoderm is ablated. It is to be expected that this ablation only modestly effects elongation.

In the genetic ablation of spinal cord cells in Figure 7, how early can one detect a shortened body axis? What percent of spinal cord cells undergo apoptosis?

© 2025. Published by The Company of Biologists under the terms of the Creative Commons Attribution License (https://creativecommons.org/licenses/by/4.0/).

Minor comments:

The last sentence of page sixteen does not logically follow from the preceding sentences, "Therefore, the redistribution of mesoderm progenitors towards and neural fate cannot explain the proportional regulation in the tail that we observe in spinal cord ablations."

Labels are missing for figure 4 G and H.

Significance

The study addresses a fundamental aspect of vertebrate development: how the body elongates and how are the proportion of neural and mesodermal tissues balanced during elongation.

Reviewer 2

Evidence, reproducibility and clarity
The manuscript by Saunders et al. investigates the axial elongation of the zebrafish embryo, focusing on interactions between spinal cord and paraxial mesoderm that coordinate proportional elongation. The authors use optical and genetic methods to ablate cells in the spinal cord during axial elongation, which causes a shortening of the spinal cord, and interestingly a shortening also of the adjacent paraxial mesoderm. The authors provide data ruling out some possible mechanisms to explain the shortening of the paraxial mesoderm in response to shortening of the spinal cord. The manuscript gives additional data to the complex process of axis elongation during vertebrate body plan formation and provides important insights into the regulation of proportional elongation during embryogenesis.

Major comments:

1. The manuscript implies in the introduction and discussion that the shortening of the paraxial mesoderm in response to shortening of the spinal cord is likely due to mechanical coupling between the two tissues. A major alternative explanation is there is signaling from the spinal cord to the paraxial mesoderm inducing extension and/or proliferation. This model is unexplored in the manuscript. Is there a change in cell number in the paraxial mesoderm? The lab has also shown nicely in a previous publication the cell behaviors associated with zebrafish paraxial mesoderm extension, which are not analyzed in this manuscript.
2. After spinal cord ablation, it is unclear whether at the end of somitogenesis the spinal cord still has fewer cells than unmanipulated embryos, or if there has been a recovery in cell number (full or partial). If there is a recovery, where are those cells coming from?
3. In figure 2E, the lateral slice image appears to show caspase 3 activation in the posterior and ventral regions of the tailbud corresponding to NMC and mesoderm progenitor regions. If so, this could contribute to shortening of the paraxial mesoderm. Related to this, does the shortened paraxial mesoderm have the equivalent number of cells compared to unmanipulated controls (similar to previous comment above)?
4. Related to the previous comment, how far away are ablations made with respect to the NMC cells (tbxta+/sox2+)? If there is unablated spinal cord between the site of ablation and the NMC cells, the NMC cells may physically not be able to participate in recovery, or may not receive a signal (physical or molecular) that there is a deficit in spinal cord.
5. A conclusion of the manuscript is that sox2 and tbxta expression is robust to spinal cord ablation, and this along with other data, is used to suggest that NMC cells do not play a role in the observed phenotypes. However, the authors also show data sox2/tbxta co-expressing cells are reduced in number after spinal cord ablation - indicating a reduction of NMC cells, which in theory could contribute to a shortening of spinal cord and paraxial mesoderm (in a proportional manner).
6. The ablation of mesoderm progenitors without a resulting shorting of the paraxial mesoderm is an interesting result. Is there a recovery in cell number? Or does the tissue show a decreased cell number/volume while still elongating to the normal length?
7. With the cell tracking experiments, as shown and referenced, the NMC cells have a collective posterior migration. My interpretation of this regarding neural contribution is that some of these cells slow their migration relative to their neighbors as they join the spinal cord. Are there any changes in migration speed (overall or in a subset of cells) that might indicate a change in the

© 2025. Published by The Company of Biologists under the terms of the Creative Commons Attribution License (https://creativecommons.org/licenses/by/4.0/).

number of cells joining the spinal cord?
8. For the analysis of cell fate switching from mesoderm to neural fate the tbx16 reporter was used. However, when cells express tbx16 they have committed or are actively committing to a mesoderm fate and are no longer NMC cells. For instance, a complete block in Wnt signaling at this point does not induce neural fate. I think it would be more appropriate to count the number of cells exiting into the mesodermal or neural territory to determine whether mesoderm fated NMC cells are switching to a neural fate in order to conclude that there is not a redistribution of NMC cells as a compensatory measure. Nevertheless, if this were the case (redistribution) I expect that it would not be able to fully compensate for a large and sudden loss of neural progenitors, but more likely would serve as a more subtle mechanism of proportional cell distribution in response to small changes in cell number/elongation.

Minor comments:

1. In figure 1E, the yellow on white color combination makes it hard to visualize where the specific staining is. The activated caspase 3 staining should be changed to a different color.
2. In figure 4, the G and H labels are missing from the figure panels.
3. The TCF::GFP transgenic line in the results text should be referred in a way more similar to the actual transgene name referred to in the methods (or provide a little more context about this line in the results).
4. The funding statement is incomplete.

Significance

General assessment:
The strength of the manuscript lies in the core finding that ablation of neural progenitors during axial elongation, which causes a shortening of the spinal cord, results also in a shortening of the adjacent paraxial mesoderm. This shows a proportional regulation of the paraxial mesoderm elongation caused by the shortening of the spinal cord and provides insight into how embryos develop a proportional body plan.

Advance:
Previous studies have examined proportional regulation at earlier stages of development and in some other specific contexts, but little is known about proportional regulation at this critical stage of development when the body axis is elongating. Thus, this study begins to fill a major gap in our understanding of proportional size regulation during vertebrate embryonic development.

Audience:
This study will be of interest to developmental biologists and stem cell biologists, as well as those studying mechanobiology.

Reviewer 3

Evidence, reproducibility and clarity

Summary:

In this study, the authors have used a laser ablation strategy to evaluate whether, and how, post gastrulation stage zebrafish embryos regulate posterior elongation in response to a change in tissue size. Using 2-photon ablation of cells in the 14-somite stage tailbud the authors show that the post gastrulation zebrafish embryo does indeed have some capacity to size regulate the proportions of tail tissue. They find that ablation of a "neural fated" region of the tailbud results in shortening of both neural and mesodermal tissues during tail extension, while ablations of the "mesoderm fated" region do not result in reduction of neural tissue or body length during tail extension. From this, they conclude that the neural tissue plays the central role in regulating size and proportion. Further, the authors characterize cell proliferation, gene expression, cell movement trajectories and Wnt signaling post-ablation, all of which indicate that mesoderm-fated cells in the neural-mesoderm competent domain do not shift/switch toward neural fates to compensate for loss of

© 2025. Published by The Company of Biologists under the terms of the Creative Commons Attribution License (https://creativecommons.org/licenses/by/4.0/).

neural cells. Finally, the authors use genetic ablation of a subset of spinal cord neurons as a secondary route to evaluate the consequences of reducing the size of the entire neural domain.

Major comments:

The authors' central experimental approach is 2-photon ablation of cells in the tailbud. Figure 1 convincingly demonstrates their ability to cleanly kill cells and to target a precise domain. Using this approach, they convincingly demonstrate that the post gastrulation zebrafish embryo does indeed have some capacity to size regulate the proportions of tail tissue.

The first set of experiments (analyzed in Fig. 2) relies on selection of neural-fated cells for ablation, which are recognized based on their expression of Sox2. This is a key experiment to show that it is the neural-fated progenitors that direct tissue size proportionality in tail elongation. One problem with this approach is that we are not shown the precise locations of the four different ablation domains (1-4), nor whether these ablations extend into the domain where tbxta is expressed - i.e. the NMC. However, Fig 3E reveals that double labeled (sox2/tbxta +ve) cells are reduced in number by the ablation, implying that even the smallest ablations do extend into the NMC domain, and thus that mesoderm progenitors are potentially being ablated in addition to neural progenitors. This makes it hard to be confident that only 'neural fated' cells are being removed. The whole experiment needs better explanation, with more clarity on the 4 different ablation domains. Ultimately, only the size 1 and 2 ablations lead to proper proportional regulation, a point that should be emphasized because it reveals that proportional regulation is limited in capacity, but which may also reflect differences in the position of the ablation rather than just the size. Related to this, the varying extents of the ablated neural regions (size 1, 2, 3, 4) should be schematized in Fig. 2A, and the same is true for the later mesoderm ablations (size 1, 2, 3), schematized in Fig 6A.

There are several aspects of Figure 2 that are unclear. In Fig. 2C, how is the "length of the spinal cord" - apparently indicated by the left white line - established? What landmark is being used to determine the end of that line? Normalizing to somite number to take minor differences in developmental timing into account is appropriate, but exactly how was that done? We need a better description of relative spinal cord /mesoderm length and its units in Fig. 2 G, H, K.

Fig. 3 investigates potential alterations in expression of sox2 and either tbxta or tbx16 expression in response to small (size 1) ablations. We see a rapid return to normal expression patterns, suggesting these domains are indeed robust to ablation. However, why are similar experiments not performed with the larger size 2 ablations, which also show regulation of body size?

Following on there is a series of experiments - Figs 3E-J, 4 and 5 - that address potential post ablation changes in cell division rates, cell migration and fate shifts, and Wnt signaling. These quite extensive data indicate that NMC cells do not increase division rates, nor do they shift fate to rebalance proportions. Overall, the data support the authors conclusions that there is no evidence of the NMC cells altering differentiation to regulate tissue proportions.

Mesoderm ablations (Fig. 6) show that posterior body length is unaffected by loss of up to ~14% of the tbxta-positive domain. This is in stark contrast to the outcome of ablations in the neural domain, and thus supports the authors conclusion that the neural region is the driver of proportional regulation.

Having found no evidence for changes in NMC behavior acting to regulate elongation and proportionality, the authors attempt to test the hypothesis that it is instead spinal cord morphogenesis that is driving elongation. Specifically, they predict that reduction in neural tissue anterior to the tailbud should reduce length of tail tissues. Their final experiment (Fig. 7) exploits ATS3:Kid transgenic specimens in a sophisticated approach to kill neural cells - specifically spinal cord motor neurons. However, this genetic ablation of a subset of neurons along the spinal cord is significantly different in both spatial resolution and developmental timing from the 2-photon ablation experiments, moreover the resultant phenotypes do not appear overtly similar to our eye. Overall, we find the outcome of this experiment to be somewhat equivocal: yes, the tail is shorter, but the spinal cord is actually shorter than the paraxial mesoderm - much like the largest ablations - so proportional regulation is not actually demonstrated.

© 2025. Published by The Company of Biologists under the terms of the Creative Commons Attribution License (https://creativecommons.org/licenses/by/4.0/).

We recommend the authors perform ablations of the developing neural tube anterior to the tailbud ablation domain, which would arguably test the hypothesis that anterior spinal cord morphogenesis drives tail elongation more directly than the genetic ablation experiment provided in Figure 7.

OPTIONAL: Cell tracking of the contribution of sox2+ (neural-fated), sox2+/tbxta+ (NMC), and tbxta+ (mesoderm-fated) cells starting at the 14-somite stage would strengthen conclusions regarding control of proportional elongation of the tail tissue. However, we realize that this may be more appropriate for a follow up study as it is a significant amount of work.

Our requested additional experiment uses a similar approach to the approaches shown in Figs 1, 2 and 6; it would thus seem to be realistic for the authors to complete and analyze the experiment at the same time as they are editing the text and figures to enhance clarity. We estimate this would take 2-3 months.

Reproducibility:
There are some issues here. The specific differences between regions that were ablated in the tailbud are unclear. The differences in size and position of ablated territories (in both Figures 2 and 6) are not sufficiently described to be reproducible. Number of nuclei ablated does not address precise location of the procedure. This concern could be addressed by adding size and position information (relative to anatomical landmarks) to the diagrams in Figures 2A and 6A and through additional explanation in the methods section.

The measurements collected (spinal cord length, trunk paraxial mesoderm length) for the statistical analyses in this study are not described in sufficient detail to be reproduced.

Experiments are adequately replicated and statistical analysis is adequate

Minor comments:

Images of tails resulting from Size 2 and 3 ablations in Figure 2 and Size 2 ablation in Figure 6 should be added.

Prior studies are referenced appropriately. Clarity of text and figures:
Fig 3A includes both control and ablated specimens - for clarity in the text it would be better to separate these into Figs 3A (control) and 3B (ablated).

Figure 4 is missing labels for panels G and H. Figure legend description for C) says "Reprehensive images" instead of representative images.

Suggestions to help the authors improve the presentation of their data and conclusions:

Figure 5A and B seem to fit better with the type of data presented in Figure 4. It is unclear why they were included in Figure 5.

Significance

General assessment:

To provide context, there has been a great deal of interest in the role of neuro-mesodermal progenitors (NMPs) in vertebrate body elongation in recent years. Whether and how NMP cells function in zebrafish, with their different mode of gastrulation and extension in comparison to amniotes, was for some time quite unclear. More recent data, however, much from this group and their collaborators, suggest that bipotent NMPs are not present in the elongating zebrafish, but that there is nevertheless a region that has shared competence to produce either neural or mesodermal tissue - the NMC. How the NMC precisely functions and relates to body elongation remain open questions. In addition, while it has been demonstrated that there is proportional regulation in the context of alterations before and during gastrulation, this has not been well addressed post gastrulation.

© 2025. Published by The Company of Biologists under the terms of the Creative Commons Attribution License (https://creativecommons.org/licenses/by/4.0/).

Here the authors have focused on proportional size control in the elongating zebrafish tail and the role of the NMC region. They have undertaken a direct embryological test of how proportional tail development responds to loss of key progenitor populations in the tailbud. While the ablated domains need to be clarified, the results from their experiments clearly show size regulation and also suggest that loss of neural progenitors reduces tail length and leads to proportional mesoderm size, while loss of the mesoderm does not have a similar reciprocal effect on spinal cord development. Importantly, the NMC is not the important driver of this regulation.

The relatively simple and direct experimental approach of 2-photon ablation is a primary strength of this paper. The authors have then pursued a thorough investigation of proliferation, gene expression, cell movements and Wnt signaling to show that the NMC is not the driver of this proportional development. As the results are negative, we are left with an interesting conclusion derived largely from a single experimental approach. We find the attempt to use a genetic approach to address this same question from a different angle to be less compelling.

General improvements:

Would come from more clarity in the description of the key experiments. The results and discussion text could also be clearer about the spatial and temporal distinctions between "neural-fated", "mesoderm-fated", and NMC regions used in this study. We encourage the authors to exercise caution about conflating descriptions of a position with solid fate assignments, especially given the complicated and moving nature of the NMC region in the developing zebrafish tailbud.

Advance:

This is a direct test of the contribution of neural and mesodermal cell populations to proportional tail elongation in zebrafish. It provides a conceptual advance in our understanding of how proportionality of the posterior body plan can be robustly regulated. This study builds on recent insights into how the tail elongates in different species and relates to the literature on multi-tissue interactions during tail elongation, including the Steventon group's prior study of the role of the notochord in elongation (McClaren and Steventon 2021, Development).

Potential Audience:

This is basic research of interest to those working in the areas of axis elongation, posterior development, and neuro-mesodermal cell fates. It will appeal not only to those working directly on zebrafish but also to researchers working on these same problems across multiple other species.

Our expertise:

Zebrafish gastrulation and cell type specification, nervous system development

---

Author response to reviewers' comments

1. General Statements [optional]

We thank the reviewers for their insightful and helpful comments. We believe this has greatly improved our manuscript.

2. Point-by-point description of the revisions

Reviewer #1
Evidence, reproducibility and clarity
This study uses laser ablations to study the mechanism of posterior body elongation in zebrafish. The authors ablate cells in the tailbud and examine the behavior of a bipotential population of cells (NMPs or NMCs) which can give rise to both spinal cord and paraxial mesoderm. The authors do

© 2025. Published by The Company of Biologists under the terms of the Creative Commons Attribution License (https://creativecommons.org/licenses/by/4.0/).

not detect a change in fates of the NMP cells but without long-term fate mapping, it is difficult to make firm conclusions about cell fates. That said, the authors see clear differences in phenotypes when ablating neural or mesodermal progenitors with the form producing larger effects on elongation. A final set of experiments that genetically ablates spinal cord cells also lead to a shortened body axis. Generally speaking, the hypothesis that spinal cord elongation drives posterior body elongation is supported by the data. However, in many cases, it is difficult to draw unambiguous conclusions from the data as they are presented.

We thank the reviewer for their comments that have prompted us to clarify the presentation and quantification of the data presented. We have included an additional figure with a full characterisation of the ATS3:GAL4;UAS:GFP experiment, together with additional experiments to follow the response of the TCF reporter at the RNA level.

Major comments:

In Figure 3A, on the 1hr ablation panel, it looks like the ablated region has more tbxta expression. Is this a consistent result?

This is not a consistent result. We have created supplementary figure 2A to show the variability in gene expression pattern amongst embryos of the same stage. We see variability in pattern among controls (as we have described in a previous study Toh et al., 2022) and variability among ablated embryos. As the increased *tbxta* in the ablation region is only present in 2 out of the 5 embryos, we have replaced the image in the main figure with a more representative example to avoid confusion.

To quantify whether there are conistent differences in gene expression we took the *sox2*/*tbxta* co-expressing nuclei in all embryos and made average maps of expression level at each timepoint (Fig 3D). We do not find any significant shift in the balance between *sox2* and *tbxta* within the NMC cells at any timepoint and conclude that the overall pattern of *sox2*/*tbxta* expression and the differentiation of the NMC cells is robust to cell loss. The text has been updated to describe this addition (Lines 252-274, pages 10-11).

Perhaps analyzing tbxta and sox2 expression using intronic probes to visualize nascent transcription would be a more sensitive assay for changes in NMP activity on the short time scales of these experiments.

We agree that this would be the ideal experiment however we have tried this, and it is not possible to make intronic probes against either gene. *Sox2* has no introns and *tbxta* has introns that are too short and repetitive in sequence to generate enough probes. Consequently, the best alternative method is to look at the levels of expression in the nuclei specifically, as this allows us to pick up peaks of signal associated with nascent transcription. As described above, this quantification is now present in figure 3 and Lines 252-274, pages 10-11

In Figure 3B, it looks like elevated Sox2 expression in the ventral tailbud after ablation. Wouldn't this suggest that more NMPs differentiate as mesoderm?

As mentioned above, there is an underlying heterogeneity within control embryos at the transcript level, as previously described (Toh et al., 2022). We also find this to be true for the *sox2* driven GFP protein levels. Our dataset of multiple ablated and control embryos does not show a consistent ventral expansion of sox2 expression (Supplementary Figure 2D).

The data in Figure 5C-H are not compelling. The conclusion is that there is no change in Wnt signaling. Maybe. There are no apparent changes, but what are the resolution of the data? How sensitive is the TCF::GFP transgene to changes in endogenous levels of Wnt signaling?

The data in Figure 5C-H is generated by taking the mean intensity in the reporter channel for each nuclear spot used for tracking. The timestep of the live movies is 30s, this gives a high resolution over one hour post-ablation. While the TCF::GFP transgenic has been shown to be highly responsive to both genetic and pharmacological manipulation of Wnt signalling (Moro et al., 2012) the authors only demonstrate that a change in the transgene transcription is present 3 hours after

© 2025. Published by The Company of Biologists under the terms of the Creative Commons Attribution License (https://creativecommons.org/licenses/by/4.0/).

manipulation. This could mean that we would not see a change in GFP protein during the time of live imaging. This information has been included in the text.

To investigate this further we have ablated and fixed embryos and controls at both 1hr (to be comparable with the end of live imaging) and 4hrs post ablation. We stained mRNA for the GFP transgene using hybridisation chain reaction, as changes in RNA expression are expected to be more rapid that the GFP protein levels we previously monitored by live imaging. We did not observe a difference at 1hr post ablation between control and ablated embryos in the posterior wall (Fig. 5G; arrow). At 4hrs post ablation we do not see any significant change to GFP mRNA localisation in the posterior wall (Fig. 5H; arrow), though there appears to be higher levels of GFP (and therefore TCF activity) in the notochord progenitors (Fig. 5H; asterisk). While this change in the notochord progenitors does not affect our overall conclusions it would be interesting in further work to investigate whether this is a direct consequence of ablation or whether it is a consequence of reduced elongation of the tail caused by dorsal progenitor loss. Description of these additional results are included in the text (Lines 439-450, page 17).

In figures 6E-G, are the paraxial mesoderm measurements made on the ablated side or unablated side? It is difficult to interpret the data in Figure 6 as only one side of the paraxial mesoderm is ablated. It is to be expected that this ablation only modestly effects elongation.

The paraxial mesoderm measurements are made on the ablated side. We have included a comparison of paraxial mesoderm length between ablated and unablated sides following mesoderm progenitor ablation in supplementary figure 5C. We find no significant difference between the measurements of either side of the tail.

Our intention with this experiment was to determine whether a redistribution of NMC cells away from mesodermal fate to neural fate could account for the reduction in paraxial mesoderm that we observe following neural progenitor ablation by directly reducing the number of mesodermal progenitors in a manner comparable to our reduction of neural progenitors. We find that ablations of this size do not affect elongation of the paraxial mesoderm (or the spinal cord) and therefore conclude that NMC cell redistribution cannot explain the results observed in Figure 2. We have adjusted the text regarding Figure 6 to better highlight our aim and the importance of the result in question.

In the genetic ablation of spinal cord cells in Figure 7, how early can one detect a shortened body axis? What percent of spinal cord cells undergo apoptosis?

We have fixed embryos from both a ATS3:GAL4;UAS:GFP and UAS:Kid cross, and a ATS3:GAL4;UAS:GFP in-cross at 20hpf (when the 22nd somite has formed). We do not observe any clear phenotype at this stage in the UAS:Kid cross. Additionally, not all embryos from the cross will have the ATS3:GAL4;UAS:Kid genotype. However, because the ATS3:GAL4 line also contains a UAS:GFP cassette we can group embryos from the UAS:Kid cross into those that have bright or weak GFP fluorescence in the spinal cord. From our experiments at 30hpf when the Kid phenotype in the spinal cord is clear we observe that all embryos with the phenotype have a reduced GFP expression in the spinal cord (Fig. 7). Therefore those embryos that have weak GFP fluorescence in the spinal cord at 20hpf are the most likely candidates for possessing the UAS:Kid transgene, though we cannot be sure of this without genotyping. This could suggest that the Kid toxin is active at 20hpf however we do not find a significant difference in body axis length (from the first somite to the tail tip) between these weakly fluorescent embryos and embryos from the ATS3:GAL4;UAS:GFP cross. Placing the effect of the Kid toxin on spinal cord elongation after 20hpf is consistent with the effect of Kid toxin expression in the notochord which is seen at 22hpf (Labbaf et al., 2022).

To get an estimation of the percent of spinal cord cells undergoing apoptosis we fixed and stained embryos for activated Caspase 3 as a marker for apoptosis at 30hpf. We observe both a reduction in UAS:GFP signal and higher levels of activated Caspase 3 in the spinal cord of UAS:Kid embryos compared to controls. This is particularly prominent in the region of tail bending. We took a consistent volume of the spinal cord in this region and calculated the relative number of apoptotic nuclei in both control and ablated embryos. There is variation in the amount of cell death between UAS:Kid embryos but on average around 16% of spinal cord cells are undergoing cell death in this

© 2025. Published by The Company of Biologists under the terms of the Creative Commons Attribution License (https://creativecommons.org/licenses/by/4.0/).

region at this time. Description of this result has been included in the text (Lines 550-560, pages 20-21).

Minor comments:

The last sentence of page sixteen does not logically follow from the preceding sentences, "Therefore, the redistribution of mesoderm progenitors towards and neural fate cannot explain the proportional regulation in the tail that we observe in spinal cord ablations."

We have clarified this conclusion in the text.

Labels are missing for figure 4 G and H.

These panels have been moved to supplementary figure 4 but their figure labels have now been added.

Significance
The study addresses a fundamental aspect of vertebrate development: how the body elongates and how are the proportion of neural and mesodermal tissues balanced during elongation.

Reviewer #2 (Evidence, reproducibility and clarity (Required)):

The manuscript by Saunders et al. investigates the axial elongation of the zebrafish embryo, focusing on interactions between spinal cord and paraxial mesoderm that coordinate proportional elongation. The authors use optical and genetic methods to ablate cells in the spinal cord during axial elongation, which causes a shortening of the spinal cord, and interestingly a shortening also of the adjacent paraxial mesoderm. The authors provide data ruling out some possible mechanisms to explain the shortening of the paraxial mesoderm in response to shortening of the spinal cord. The manuscript gives additional data to the complex process of axis elongation during vertebrate body plan formation and provides important insights into the regulation of proportional elongation during embryogenesis.

We thank this reviewer for their comments that have improved the clarity with which we describe the reproducibility and reliability of our ablations. In addition, we have included new data on cell counts within the PSM, changes in the size of the spinal cord and numbers of Tbx16 positive cells. We have also included a quantification of velocity decrease from our cell tracking data, and a new experiment following the long term distribution of mesodermal cells using photolablation- neither of which are altered following dorsal progenitor ablation.

Major comments:

1. The manuscript implies in the introduction and discussion that the shortening of the paraxial mesoderm in response to shortening of the spinal cord is likely due to mechanical coupling between the two tissues. A major alternative explanation is there is signaling from the spinal cord to the paraxial mesoderm inducing extension and/or proliferation. This model is unexplored in the manuscript. Is there a change in cell number in the paraxial mesoderm? The lab has also shown nicely in a previous publication the cell behaviors associated with zebrafish paraxial mesoderm extension, which are not analyzed in this manuscript.

We acknowledge that either inter-tissue signalling or tissue mechanics could be the cause of the proportional reduction in axis length. We have added this potential hypothesis into the introduction (lines 93-94, page 4) and into the discussion (line 688, page 25). We consider the focus of our paper to be demonstrating that spinal cord elongation is a key factor (but not the only factor) in correct paraxial mesoderm elongation during tail formation. Determining the mechanisms by which spinal cord elongation drives PSM elongation is a new question raised by this work that is clearly of great interest to the field.

With regard to whether there is a change in cell number in the paraxial mesoderm we have quantified the number of nuclei expressing *tbx16* positive cells following neural progenitor

© 2025. Published by The Company of Biologists under the terms of the Creative Commons Attribution License (https://creativecommons.org/licenses/by/4.0/).

ablations (supplementary figure 3D,E) and do not see a change either immediately after ablation or at 4hrs post ablation. In the previous publication mentioned by the reviewer (Thomson et al., 2021), it was demonstrated that the movement of cells in the PSM is largely random so it would be difficult to define a metric which we would expect to change following dorsal progenitor ablation.

2. After spinal cord ablation, it is unclear whether at the end of somitogenesis the spinal cord still has fewer cells than unmanipulated embryos, or if there has been a recovery in cell number (full or partial). If there is a recovery, where are those cells coming from?

We have looked at the posterior spinal cord at the end of somitogenesis and we find that it is significantly decreased in height in the dorsal ventral axis (Supplementary Figure 3A,C). We have quantified the number of nuclei within the posterior tip (from the anterior PSM to the tip of the spinal cord) of the spinal cord and find that there remains a decrease in nuclei number compared to control embryos in 2 out of 3 ablated embryos. (Supplementary Figure 3B). While this does not amount to a significant difference according to the statistical test this could be due to the small sample number and relatively large variation in nuclei number that we recover from control embryos. Redistribution of cells along the spinal cord during its elongation could mean that the decrease in cell number is spread out and below the threshold of detection for our current quantification methods. However, it is possible that there is a rescue in the number of some cells in the spinal cord. Regardless, this does not impact our broader conclusions that the loss of neural progenitors causes a decrease in axial elongation and that this is not brought about by a shifting of progenitors from mesodermal to neural fate in the tailbud. Description of this result is now in the text (Lines 303-305, page 12)

3. In figure 2E, the lateral slice image appears to show caspase 3 activation in the posterior and ventral regions of the tailbud corresponding to NMC and mesoderm progenitor regions. If so, this could contribute to shortening of the paraxial mesoderm. Related to this, does the shortened paraxial mesoderm have the equivalent number of cells compared to unmanipulated controls (similar to previous comment above)?

We first observe apoptotic cells in the neural-fated progenitor region of the tailbud, subsequently we observe activated caspase3 staining in the lateral slice. This is due to the apoptotic debris moving with the flow of the cells. Unablated cells rapidly make new cell-cell contacts (see Fig. 4) and the dying cells break down and as such they seem to flow out of the tailbud faster than the cells themselves. The debris can be seen to be moved around rapidly in live movies; an example movie has now been provided (Supplementary Movie 1). A description of this has now been included in the text (lines 153-156, page 7). An additional example can also be seen in supplementary figure 4F.

Additionally, we have quantified the number of *tbx16* positive nuclei and do not find a significant difference in nuclei number even 4hrs after dorsal progenitor ablation (Supplementary Figure 3D,E). In contrast mesoderm progenitor ablations, do cause a decrease in *tbx16* nuclei number, yet there is no decrease in paraxial mesoderm length (Fig. 6). Together this proves that the proportional regulation following neural progenitor ablation can not be explained by a mis-allocation of cells from the mesoderm to the neural lineage.

4. Related to the previous comment, how far away are ablations made with respect to the NMC cells (tbxta+/sox2+)? If there is unablated spinal cord between the site of ablation and the NMC cells, the NMC cells may physically not be able to participate in recovery, or may not receive a signal (physical or molecular) that there is a deficit in spinal cord.

Ablations are made within the dorsal-posterior region of the tailbud. This region contains both sox2+/tbxta- and sox2+/tbxta+ cells, this can be seen in the gene expression patterns in Fig. 3). This means that ablations take place within the dorsal part of the NMC region. The observation that cell debris can pass from the ablated region into the ventral tailbud suggests that there is not a significant boundary generated by the ablation.

© 2025. Published by The Company of Biologists under the terms of the Creative Commons Attribution License (https://creativecommons.org/licenses/by/4.0/).

5. A conclusion of the manuscript is that sox2 and tbxta expression is robust to spinal cord ablation, and this along with other data, is used to suggest that NMC cells do not play a role in the observed phenotypes. However, the authors also show data sox2/tbxta co-expressing cells are reduced in number after spinal cord ablation - indicating a reduction of NMC cells, which in theory could contribute to a shortening of spinal cord and paraxial mesoderm (in a proportional manner).

Sox2/*tbxta* cells are reduced in number after ablation because the ablation is performed in the dorsal region of the tailbud which contains both *sox2* only and *sox2*/*tbxta* cells (Figure 3A and B). There is clear reduction in gene expression in this dorsal region only, immediately after ablation (Fig. 3B). Previous studies have shown that this dorsal region is majority neural-fated (Kanki and Ho, 1997; Attardi et al., 2018; Lange et al., 2024), however to avoid confusion we now refer to it in the text by its location rather than its presumed fate. A reduction in NMC cells could only contribute to the shortening of the tail tissues if there was a re-distribution of progenitors from ventral to dorsal regions in the tailbud (i.e. mesodermal to neural fate) which we do not observe over short or long-time scales (Fig. 4). Additionally, direct reduction of the laterally and ventrally located mesoderm progenitors would be expected to phenocopy the effect on paraxial mesoderm which we also do not observe (Fig. 6).

6. The ablation of mesoderm progenitors without a resulting shorting of the paraxial mesoderm is an interesting result. Is there a recovery in cell number? Or does the tissue show a decreased cell number/volume while still elongating to the normal length?

We have quantified the number of *tbx16* positive cells at 4hrs following ablation and we observe a decrease in number in ablated embryos compared to controls, this is now included in Figure 6 H and I. We also observe that the debris is spread throughout the PSM in line with previous descriptions of the PSM as a fluid tissue with extensive cell rearrangement (Mongera et al., 2018; Lawton et al., 2013; Thomson et al., 2021). This could mean that the loss of cells ends up spread out along the paraxial mesoderm so that each somite has only a small reduction in cell number. We have also quantified the number of nuclei in the anterior PSM at the end of somitogenesis and find that there is no difference in number between control and ablated embryos. This could be because by this stage the reduction in cell number is less than biological and/or technical variation in PSM number or because there is a later compensatory response. The data is shown in Supplementary Figure 5. Description of these results are now included in the text (Lines 513-521, page 19).

7. With the cell tracking experiments, as shown and referenced, the NMC cells have a collective posterior migration. My interpretation of this regarding neural contribution is that some of these cells slow their migration relative to their neighbors as they join the spinal cord. Are there any changes in migration speed (overall or in a subset of cells) that might indicate a change in the number of cells joining the spinal cord?

We agree that this could be affecting spinal cord elongation. To clarify this, we took the tracks analysed in Figures 4 and calculated the difference in median velocity between the last 5 minutes of each track and the first 5 minutes of each track. This gives a normally distributed set of velocity changes centred around 0. The lower quartile of this data includes only tracks which have a negative difference in velocity and therefore have slowed down over the lifetime of the track. These tracks are uniformly distributed throughout the tailbud and between both control and ablated embryos. This suggests that over this short time scale changes to velocity are not associated with dorsal localisation of neural differentiation and ablation does not appear to affect the amount of tracks that decrease in velocity. These results are shown in supplementary figure 4E.

8. For the analysis of cell fate switching from mesoderm to neural fate the tbx16 reporter was used. However, when cells express tbx16 they have committed or are actively committing to a mesoderm fate and are no longer NMC cells. For instance, a complete block in Wnt signaling at this point does not induce neural fate. I think it would be more appropriate to count the number of

© 2025. Published by The Company of Biologists under the terms of the Creative Commons Attribution License (https://creativecommons.org/licenses/by/4.0/).

cells exiting into the mesodermal or neural territory to determine whether mesoderm fated NMC cells are switching to a neural fate in order to conclude that there is not a redistribution of NMC cells as a compensatory measure. Nevertheless, if this were the case (redistribution) I expect that it would not be able to fully compensate for a large and sudden loss of neural progenitors, but more likely would serve as a more subtle mechanism of proportional cell distribution in response to small changes in cell number/elongation.

We agree with the reviewer that tbx16 represents mesoderm commitment so the use of the tbx16 reporter would not be expected to give us any insight into whether there are changes in the distribution of fates within the NMC cells. Instead we include this data to show that there is no breakdown in the normal progression of mesoderm differentiation or any committed mesodermal cells entering the NMC domain. We have clarified this in the text and moved this panel to supplementary figure 2E.

With regard to the cells exiting into neural or mesodermal territory we have performed a relevant quantification by looking at the direction of displacement of our live imaging tracks (Fig 5B now Fig4). The result demonstrates that there is not a large movement of cells from the ventral, mesodermal territory into the dorsal neural territory during the short-term of ablation healing. However, this raises the question of what happens to the long-term behaviour of the progenitors. To address this, we performed a photolabeling experiment to trace how the movement of ventral and lateral progenitors is effected by dorsal progenitor ablation. We find that no labelled cells in either control or ablated embryos end up in the spinal cord showing that there is no large-scale later shift in progenitor flow. This data is now included in Figure 4.
Description of this result is included in the text (Lines 384-397, page 15).

However, we agree that there is unlikely to be a strict barrier between dorsally and ventrally located NMC cells so there could be a small amount of NMC cell redistribution which is not picked up by our analyses and. As noted, what is important for our argument is that any redistribution of the NMC cells will not be *compensatory* and account for the reduction in paraxial mesoderm length, given that direct ablation of mesoderm-fated progenitors does not affect the elongation. We have clarified in the discussion that we can specifically rule out a widespread/large-scale re-distribution of NMC Cells (Lines 664-672,page 25). While we can't rule out that some cells do undergo a change, if they do, it does not appear to be important for proportional regulation of the tissues.

Minor comments:

1. In figure 1E, the yellow on white color combination makes it hard to visualize where the specific staining is. The activated caspase 3 staining should be changed to a different color.
This has been changed.

2. In figure 4, the G and H labels are missing from the figure panels.
This has been changed.

3. The TCF::GFP transgenic line in the results text should be referred in a way more similar to the actual transgene name referred to in the methods (or provide a little more context about this line in the results).
The results have been updated to include more context about the transgene.

4. The funding statement is incomplete.
This has been completed.

Reviewer #2 (Significance (Required)):

General assessment: The strength of the manuscript lies in the core finding that ablation of neural progenitors during axial elongation, which causes a shortening of the spinal cord, results also in a shortening of the adjacent paraxial mesoderm. This shows a proportional regulation of the paraxial mesoderm elongation caused by the shortening of the spinal cord and provides insight into how embryos develop a proportional body plan.

© 2025. Published by The Company of Biologists under the terms of the Creative Commons Attribution License (https://creativecommons.org/licenses/by/4.0/).

Advance: Previous studies have examined proportional regulation at earlier stages of development and in some other specific contexts, but little is known about proportional regulation at this critical stage of development when the body axis is elongating. Thus, this study begins to fill a major gap in our understanding of proportional size regulation during vertebrate embryonic development.

Audience: This study will be of interest to developmental biologists and stem cell biologists, as well as those studying mechanobiology.

Reviewer #3 (Evidence, reproducibility and clarity (Required)):

Summary:

In this study, the authors have used a laser ablation strategy to evaluate whether, and how, post gastrulation stage zebrafish embryos regulate posterior elongation in response to a change in tissue size. Using 2-photon ablation of cells in the 14-somite stage tailbud the authors show that the post gastrulation zebrafish embryo does indeed have some capacity to size regulate the proportions of tail tissue. They find that ablation of a "neural fated" region of the tailbud results in shortening of both neural and mesodermal tissues during tail extension, while ablations of the "mesoderm fated" region do not result in reduction of neural tissue or body length during tail extension. From this, they conclude that the neural tissue plays the central role in regulating size and proportion. Further, the authors characterize cell proliferation, gene expression, cell movement trajectories and Wnt signaling post-ablation, all of which indicate that mesoderm- fated cells in the neural-mesoderm competent domain do not shift/switch toward neural fates to compensate for loss of neural cells. Finally, the authors use genetic ablation of a subset of spinal cord neurons as a secondary route to evaluate the consequences of reducing the size of the entire neural domain.

We thank this reviewer for their insightful comments and agree that directly assigning cell fate to location in the tailbud is adding an inference to the description of our experiments. We now refer to the ablation regions in terms of their location, and reference fate mapping studies as an inference of their fate contributions and draw our conclusions with reference to these studies.
Nevertheless, the accumulation of observations in the paper rules out the possibility that the compensation of dorsal progenitors by ventral ones, or through the rescue of NMC progenitor numbers. We have also included additional experiments ablating spinal cord regions as requested by this reviewer.

Major comments:

The authors' central experimental approach is 2-photon ablation of cells in the tailbud. Figure 1 convincingly demonstrates their ability to cleanly kill cells and to target a precise domain. Using this approach, they convincingly demonstrate that the post gastrulation zebrafish embryo does indeed have some capacity to size regulate the proportions of tail tissue.

The first set of experiments (analyzed in Fig. 2) relies on selection of neural-fated cells for ablation, which are recognized based on their expression of Sox2. This is a key experiment to show that it is the neural-fated progenitors that direct tissue size proportionality in tail elongation. One problem with this approach is that we are not shown the precise locations of the four different ablation domains (1-4), nor whether these ablations extend into the domain where tbxta is expressed - i.e. the NMC.

We agree that this was not previously clear in the text or figures. We have added example images of all ablation types into Supplementary Figures 1, 5, and 7. Additionally, we have clarified in the text that all these ablations extend into the NMC domain and will include a mixture of *sox2+/tbxta-* and *sox2+/tbxta+* cells (line 145). However, based on previous fate mapping and lineage tracing we can be confident that the majority of cells in the ablated region, the dorsal aspect of the tailbud, would normally contribute to spinal cord, though we acknowledge that there may be a few mesodermal-fated progenitors ablated. However, given that we observe a completely different response when directly ablate mesodermal progenitors we can conclude that the effect we see from dorsal progenitor ablations is due to the loss of neural progenitor (either *sox2* only or NMC)

© 2025. Published by The Company of Biologists under the terms of the Creative Commons Attribution License (https://creativecommons.org/licenses/by/4.0/).

cells.

As the reviewer notes below we must be careful when dealing with domains of general fate, morphological locations and gene expression which each have a complex relationship with each other in the tailbud over the course of development. Consequently, we have opted to change mention in the text of neural-fated progenitors and instead refer to them as dorsal progenitors based on their location in the tailbud. We include discussion on this point in the text (lines 113-123, page 5) and a schematic for clarification (Fig. 1A).

However, Fig 3E reveals that double labeled (sox2/tbxta +ve) cells are reduced in number by the ablation, implying that even the smallest ablations do extend into the NMC domain, and thus that mesoderm progenitors are potentially being ablated in addition to neural progenitors.

We are ablating NMC cells and these are located in the region of the tailbud that contributes to spinal cord. While we can't guarantee that there are no cells in this region that might have contributed to mesodermal tissue they will be in a minority as previous fate mapping studies have shown that this is a majority neural-fated region (Kanki and Ho, 1997; Attardi et al., 2018; Lange et al., 2024). Importantly, we then ablate laterally located mesoderm-fated progenitors (which will include some laterally located *sox2+/tbxta+* cells as well *sox2-/tbxta+* ones) and find that there is no effect on paraxial mesoderm elongation. This suggests that even if we are ablating some mesoderm-fated progenitors in our neural progenitor ablations, mesoderm progenitor loss at this scale cannot be the cause of reduced paraxial mesoderm and spinal cord length. We have made sure to clarify this point in the text and as noted above now refer to the ablations based on their location rather than the presumed fate of the progenitors.

This makes it hard to be confident that only 'neural fated' cells are being removed. The whole experiment needs better explanation, with more clarity on the 4 different ablation domains.
Ultimately, only the size 1 and 2 ablations lead to proper proportional regulation, a point that should be emphasized because it reveals that proportional regulation is limited in capacity, but which may also reflect differences in the position of the ablation rather than just the size.
Related to this, the varying extents of the ablated neural regions (size 1, 2, 3, 4) should be schematized in Fig. 2A, and the same is true for the later mesoderm ablations (size 1, 2, 3), schematized in Fig 6A.

We agree that showing the locations of all the ablations is important and have included example images in Supplementary Figures 1, 5, and 7. The text has been updated to emphasise the limit to proportional regulation (line 209,page8). We have also updated the text to clarify the morphological criteria that are used to specify the ablation location (line 144, page 6). We ablate cells in an arc at the midline of the tailbud which is dorsal and posterior to the notochord progenitors which are a morphologically clear feature of the tailbud. Our mapping of a selection of ablations in Fig. 1 shows that a practiced user has a high precision in localising these ablations. In addition, we created supplementary figure 1 to highlight how the different ablation sizes were achieved. The method for increasing ablation size is first to increase the z-depth of the ablation while remaining within the neural progenitors. Subsequently, the largest ablations do extend more anteriorly towards the neural tube. A detailed explanation of this is included in the materials and methods (lines 722-746,pages 27-28).

While the loss of proportional regulation could be due to the ablation of certain cells not captured at the small ablation sizes it is clear that increased loss of progenitors generally leads to a decrease in the elongation of the structures they would normally form (Fig. 2, 6). As noted above we now name the ablations based on their location in the tailbud rather than the presumed fate of the progenitors.

There are several aspects of Figure 2 that are unclear. In Fig. 2C, how is the "length of the spinal cord" - apparently indicated by the left white line - established? What landmark is being used to determine the end of that line? Normalizing to somite number to take minor differences in developmental timing into account is appropriate, but exactly how was that done? We need a better description of relative spinal cord /mesoderm length and its units in Fig. 2 G, H, K.

We agree that this was not clear in the text. The spinal cord length was measured from 22nd somite

© 2025. Published by The Company of Biologists under the terms of the Creative Commons Attribution License (https://creativecommons.org/licenses/by/4.0/).

to the tip of the spinal cord. At these stages the tip of the spinal cord can be determined based on where the lumen ends. The measurements were made along the floor plate. The mesoderm length was measured from 22nd somite to the medial tip of the tailbud as by this stage only mesoderm cells remain in the tailbud. The 22nd somite is posterior to the yolk extension and always within the tail. Also, fate mapping shows that cells in the dorsal-posterior wall of the early tailbud contribute spinal cord and paraxial mesoderm up to the 22nd somite (Attardi et al., 2018). The length measurements were normalised by dividing by the total somite number for the embryo so the units are microns per somite. The text has been updated to clarify these points and the units have been added to the figure legends. A detailed description of tail length measurements can now be found in the materials and methods (Lines 808-829, pages 30-31).

Fig. 3 investigates potential alterations in expression of sox2 and either tbxta or tbx16 expression in response to small (size 1) ablations. We see a rapid return to normal expression patterns, suggesting these domains are indeed robust to ablation. However, why are similar experiments not performed with the larger size 2 ablations, which also show regulation of body size?

The primary reason that similar experiments were not performed with size 2 ablations is that these sized ablations are closer to the threshold at which proportional regulation is lost. Given that there is likely some variation in the amount of cell death induced by the laser, as well as slight variation in exact ablation size and location, we did not want to add the confounding possibility that the data could include embryos that are not proportionally regulating. We have performed some size 2 ablations and fixed them at 4hrs post fertilisation to examine the expression patterns, examples are included in Supplementary Fig 2. Consistent with the smaller ablations we see that there remains *sox2*/*tbxta* co-expression in the posterior wall but it appears to be weaker than control embryos. An additional reason not to use larger ablation sizes is that these expression patterns are harder to analyse because the greater levels of cell death cause characteristic circular spikes in gene expression (see arrow) that are associated with pyknotic nuclei.

Following on there is a series of experiments - Figs 3E-J, 4 and 5 - that address potential post ablation changes in cell division rates, cell migration and fate shifts, and Wnt signaling. These quite extensive data indicate that NMC cells do not increase division rates, nor do they shift fate to rebalance proportions. Overall, the data support the authors conclusions that there is no evidence of the NMC cells altering differentiation to regulate tissue proportions.

Mesoderm ablations (Fig. 6) show that posterior body length is unaffected by loss of up to ~14% of the tbxta-positive domain. This is in stark contrast to the outcome of ablations in the neural domain, and thus supports the authors conclusion that the neural region is the driver of proportional regulation.

Having found no evidence for changes in NMC behavior acting to regulate elongation and proportionality, the authors attempt to test the hypothesis that it is instead spinal cord morphogenesis that is driving elongation. Specifically, they predict that reduction in neural tissue anterior to the tailbud should reduce length of tail tissues. Their final experiment (Fig. 7) exploits ATS3:Kid transgenic specimens in a sophisticated approach to kill neural cells - specifically spinal cord motor neurons. However, this genetic ablation of a subset of neurons along the spinal cord is significantly different in both spatial resolution and developmental timing from the 2-photon ablation experiments, moreover the resultant phenotypes do not appear overtly similar to our eye. Overall, we find the outcome of this experiment to be somewhat equivocal: yes, the tail is shorter, but the spinal cord is actually shorter than the paraxial mesoderm - much like the largest ablations - so proportional regulation is not actually demonstrated.

We recommend the authors perform ablations of the developing neural tube anterior to the tailbud ablation domain, which would arguably test the hypothesis that anterior spinal cord morphogenesis drives tail elongation more directly than the genetic ablation experiment provided in Figure 7.

The use of the genetic ablation technique is important for two reasons, one is that it provides an alternative method to validate the effects of spinal cord cell or progenitor loss on the elongation of the tail, and the other is that large ablations using 2-photon often generate a large clump of cellular debris that effects the ability to properly understand the phenotype whereas this method causes widespread cell death that is distributed throughout the spinal cord. However, we

© 2025. Published by The Company of Biologists under the terms of the Creative Commons Attribution License (https://creativecommons.org/licenses/by/4.0/).

acknowledge that further details are needed to back up our conclusions, so we have performed several experiments to address the above concerns:

Firstly, we fixed ATS3:GAL4;UAS:GFP;UAS:Kid and ATS3:GAL4;UAS:GFP embryos at 20hpf and demonstrate that body elongation is not yet significantly different between control embryos and those from the UAS:Kid cross that exhibit weaker GFP fluorescence (Supplementary Fig. 6),which at later stages is associated with the ATS3:Kid phenotype (Fig. 7A). This suggests that most of the effect on spinal cord and paraxial mesoderm elongation that we observe occurs during tail elongation.

Secondly, we perform ablations of the trunk spinal cord anterior to the pre-somitic mesoderm at the 14 somite stage. Morphologically these embryos have a small dorsal bending. We observe no effect on tissue length or proportions in the tail but larger trunk spinal cord ablations do cause a decrease in the length of adjacent paraxial mesoderm. This demonstrates that though larger ablation sizes are required in the trunk compared to the tailbud, perturbation of spinal cord cell number effects the elongation of the paraxial mesoderm along the body axis. This is likely to be a proportional effect as presumably the spinal cord is itself shorter in this region but in the absence of morphological landmarks we cannot quantify this. This is now included in supplementary figure 7.

Finally, as suggested we perform ablations of the tail spinal cord anterior to the tailbud at 14 somite stage. Morphologically these embryos exhibit a clear dorsal bending. We find that ablations of the spinal cord in the tail cause a decrease in spinal cord and paraxial mesoderm elongation that is most obvious in the anterior of the paraxial mesoderm. However, similar to the genetic ablations this affect on elongation is not proportional. This also demonstrates the role for spinal cord elongation as a factor in paraxial mesoderm elongation but highlights that proportional regulation does not occur in all regions of the body axis. This data is now included in figure 8. Description of the additional results presented in figure 8 and supplementary figure 7 are included in the text (lines 599-625, pages 22-23).

OPTIONAL: Cell tracking of the contribution of sox2+ (neural-fated), sox2+/tbxta+ (NMC), and tbxta+ (mesoderm-fated) cells starting at the 14-somite stage would strengthen conclusions regarding control of proportional elongation of the tail tissue. However, we realize that this may be more appropriate for a follow up study as it is a significant amount of work.

At present, there are no available reporter lines that accurately label sox2 and tbxta co- expressing cells in the tailbud, making a genetic lineage tracing approach unfeasible. As an alternative, we have tracked cell movements in the tailbud by live imaging to determine if cells are moving dorsally to replace those cells upon ablation. We have included an additional experiment here using photo-ablation to follow ventral NMCs over a longer time period. The key prediction of our model is that ventral NMCs in the tailbud should not contribute to the spinal cord upon ablation of dorsal NMCs. To test this, we labelled the ventrally located mesodermal fated progenitors and then performed neural progenitor ablations on half of them. We do not find any contribution from mesodermal cells into the neural tube demonstrating that there is no shift of progenitors in the tailbud. This data is shown in figure 4 and description of this result is included in the text (Lines 384-397, page 15).

It is worth noting here that gene expression is also not a reliable marker for cell fate within the tailbud, given the heterogeneous expression observed for sox2 and tbxta in stage matched embryos (Toh et al., 2021). Therefore, a lineage tracing study based on gene expression reporters would not give us further insight into changes in cell fate.

Our requested additional experiment uses a similar approach to the approaches shown in Figs 1, 2 and 6; it would thus seem to be realistic for the authors to complete and analyze the experiment at the same time as they are editing the text and figures to enhance clarity. We estimate this would take 2-3 months.

We have performed this experiment as requested and now include this data in an additional figure (Figure 8).

© 2025. Published by The Company of Biologists under the terms of the Creative Commons Attribution License (https://creativecommons.org/licenses/by/4.0/).

Reproducibility:
There are some issues here. The specific differences between regions that were ablated in the tailbud are unclear. The differences in size and position of ablated territories (in both Figures 2 and 6) are not sufficiently described to be reproducible. Number of nuclei ablated does not address precise location of the procedure. This concern could be addressed by adding size and position information (relative to anatomical landmarks) to the diagrams in Figures 2A and 6A and through additional explanation in the methods section.

We have included representative examples of ablated regions for all ablation sizes so the localisation of the ablations can be visualised in Supplementary figures 1, 5, and 7. Ablation size information is already included in Figures 2 and 6. A complete description of how the different ablations were localised is now described in both the main text and the materials and methods. In figure 1C we demonstrate that a practiced user has a high precision of ablations in the dorsal progenitor region by mapping together a group of ablations.

The measurements collected (spinal cord length, trunk paraxial mesoderm length) for the statistical analyses in this study are not described in sufficient detail to be reproduced.
The method has now been elaborated on both in the main results and in the materials and methods.

Experiments are adequately replicated and statistical analysis is adequate

Minor comments:

Images of tails resulting from Size 2 and 3 ablations in Figure 2 and Size 2 ablation in Figure 6 should be added.

We have added these images into Supplementary Figures 1 and 5.

Prior studies are referenced appropriately.

Clarity of text and figures:

Fig 3A includes both control and ablated specimens - for clarity in the text it would be better to separate these into Figs 3A (control) and 3B (ablated).

This has been corrected in the text and the figure.

Figure 4 is missing labels for panels G and H. Figure legend description for C) says "Reprehensive images" instead of representative images.

This has been corrected.

Suggestions to help the authors improve the presentation of their data and conclusions:

Figure 5A and B seem to fit better with the type of data presented in Figure 4. It is unclear why they were included in Figure 5.

This has been changed. Figure 5A/B have been moved to Fig 4 and some parts of Fig 4 have been moved to Supplementary Figure 4.

Reviewer #3 (Significance (Required)):

General assessment:

To provide context, there has been a great deal of interest in the role of neuro-mesodermal progenitors (NMPs) in vertebrate body elongation in recent years. Whether and how NMP cells function in zebrafish, with their different mode of gastrulation and extension in comparison to amniotes, was for some time quite unclear. More recent data, however, much from this group and their collaborators, suggest that bipotent NMPs are not present in the elongating zebrafish, but that there is nevertheless a region that has shared competence to produce either neural or

© 2025. Published by The Company of Biologists under the terms of the Creative Commons Attribution License (https://creativecommons.org/licenses/by/4.0/).

mesodermal tissue - the NMC. How the NMC precisely functions and relates to body elongation remain open questions. In addition, while it has been demonstrated that there is proportional regulation in the context of alterations before and during gastrulation, this has not been well addressed post gastrulation.

Here the authors have focused on proportional size control in the elongating zebrafish tail and the role of the NMC region. They have undertaken a direct embryological test of how proportional tail development responds to loss of key progenitor populations in the tailbud. While the ablated domains need to be clarified, the results from their experiments clearly show size regulation and also suggest that loss of neural progenitors reduces tail length and leads to proportional mesoderm size, while loss of the mesoderm does not have a similar reciprocal effect on spinal cord development. Importantly, the NMC is not the important driver of this regulation.

The relatively simple and direct experimental approach of 2-photon ablation is a primary strength of this paper. The authors have then pursued a thorough investigation of proliferation, gene expression, cell movements and Wnt signaling to show that the NMC is not the driver of this proportional development. As the results are negative, we are left with an interesting conclusion derived largely from a single experimental approach. We find the attempt to use a genetic approach to address this same question from a different angle to be less compelling.

General improvements:

Would come from more clarity in the description of the key experiments. The results and discussion text could also be clearer about the spatial and temporal distinctions between "neural-fated", "mesoderm-fated", and NMC regions used in this study. We encourage the authors to exercise caution about conflating descriptions of a position with solid fate assignments, especially given the complicated and moving nature of the NMC region in the developing zebrafish tailbud.

We agree that the various properties of individual or even groups of cells within the tailbud, such as their fate, gene expression state and location have complex relationships with one another and are both heterogenous in space and dynamic over time. To help with clarity in this issue we have changed the way we refer to ablations in the text and instead of using the criteria of fate we use the location of the ablated progenitors.

Advance:

This is a direct test of the contribution of neural and mesodermal cell populations to proportional tail elongation in zebrafish. It provides a conceptual advance in our understanding of how proportionality of the posterior body plan can be robustly regulated. This study builds on recent insights into how the tail elongates in different species and relates to the literature on multi- tissue interactions during tail elongation, including the Steventon group's prior study of the role of the notochord in elongation (McClaren and Steventon 2021, Development).

Potential Audience:

This is basic research of interest to those working in the areas of axis elongation, posterior development, and neuro-mesodermal cell fates. It will appeal not only to those working directly on zebrafish but also to researchers working on these same problems across multiple other species.

Our expertise:

Zebrafish gastrulation and cell type specification, nervous system development

---

Original submission

First decision letter

© 2025. Published by The Company of Biologists under the terms of the Creative Commons Attribution License (https://creativecommons.org/licenses/by/4.0/).

MS ID#: DEVELOP/2024/204438

MS TITLE: Spinal Cord Elongation Enables Proportional Regulation of the Zebrafish Posterior Body

AUTHORS: Dillan Saunders; Carlos Camacho de la Macorra; Benjamin Steventon
ARTICLE TYPE: Review Commons Transfer

Thank you for sending your manuscript to Journal of Cell Science through Review Commons.

I am happy to tell you that your manuscript has been accepted for publication in Development, pending our standard publication integrity checks.

Comments from the Reviewers:

Reviewer 1:

This manuscript has been significantly improved since the first round of review (at Review Commons). As before, the strength of the manuscript lies in the core finding of a proportional regulation of paraxial mesoderm elongation in response to shortening of the spinal cord, which provides insight into how embryos develop a proportional body plan. Previous studies have examined proportional regulation at earlier stages of development and in some other specific contexts, but little is known about proportional regulation at this critical stage of development when the body axis is elongating. Thus, this study makes inroads to a major gap in our understanding of proportional size regulation during vertebrate embryonic development.

The authors have responded well to the diverse comments from multiple reviewers and are to be applauded for their thorough quantitative analyses. It is my opinion that this manuscript represents an interesting advance and is now ready for publication.

Reviewer 2:

The authors have done extensive revisions, including additional experiments and analysis, to satisfy my original concerns.

© 2025. Published by The Company of Biologists under the terms of the Creative Commons Attribution License (https://creativecommons.org/licenses/by/4.0/).
